# Tuning G-Quadruplex Nanostructures with Lipids. Towards Designing Hybrid Scaffolds for Oligonucleotide Delivery †

**DOI:** 10.3390/ijms22010121

**Published:** 2020-12-24

**Authors:** Santiago Grijalvo, Anna Clua, Marc Eres, Raimundo Gargallo, Ramon Eritja

**Affiliations:** 1 Institute for Advanced Chemistry of Catalonia (IQAC-CSIC), Jordi Girona 18-26, E-08034 Barcelona, Spain; sgrgma@cid.csic.es (S.G.); acvtnt@cid.csic.es (A.C.); marcuseres@gmail.com (M.E.); 2 Networking Center on Bioengineering, Biomaterials and Nanomedicine (CIBER BBN), Jordi Girona 18-26, E-08034 Barcelona, Spain; 3 Department of Chemical Engineering and Analytical Chemistry, University of Barcelona, Martí i Franquès 1-11, E-08028 Barcelona, Spain; raimon_gargallo@ub.edu

**Keywords:** antisense oligonucleotides, circular dichroism, G-quadruplex, gene delivery, gene transfection, lipids, luciferase, nucleic acid conjugates, solid-phase, solution-phase

## Abstract

Two G-quadruplex forming oligonucleotides [d(TG_4_T)_4_ and d(TG_6_T)_4_] were selected as two tetramolecular quadruplex nanostructures because of their demonstrated ability to be modified with hydrophobic molecules. This allowed us to synthesize two series of G-quadruplex conjugates that differed in the number of G-tetrads, as well as in the terminal position of the lipid modification. Both solution and solid-phase syntheses were carried out to yield the corresponding lipid oligonucleotide conjugates modified at their 3′- and 5′-termini, respectively. Biophysical studies confirmed that the presence of saturated alkyl chains with different lengths did not affect the G-quadruplex integrity, but increased the stability. Next, the G-quadruplex domain was added to an 18-mer antisense oligonucleotide. Gene silencing studies confirmed the ability of such G-rich oligonucleotides to facilitate the inhibition of target *Renilla* luciferase without showing signs of toxicity in tumor cell lines.

## 1. Introduction

G-quadruplexes are nucleic acid structures constituted by two or more G-quartet motifs held together by Hoogsteen and π-π stacking interactions. In addition, these structures are stabilized by monovalent cations (Na^+^ and K^+^, mainly). G-quadruplexes may be parallel, antiparallel or even hybrid structures that differ according to the type of loops and the G-tetrad strands orientation [1]. G-quadruplexes were observed for the first time in the early 1900s and they were characterized by x-ray diffraction in the sixties [2]. Nowadays, applications based on G-quadruplex are many and diverse, ranging from diagnostic tools, biosensing and medicine [3,4,5]. In addition G-quadruplex structures have been visualized in human cells [6], being present at the end of the chromosomes (telomeres) as well as in some regions of the genome like 5′-UTR regions and oncogene promoters (e.g., *c-kit*, *bcl-2* and *c-myc*), emerging as attractive targets for cancer therapy [5]. In this regard, human chromosomal DNA contains single repeated guanine-rich residues (TTAGGG) at the telomeres. These areas have variable lengths, controlled by telomerases, which diminish with the number of cell divisions. In contrast, cancer cells have long telomeres providing uncontrolled cellular proliferation [7,8]. Therefore, appropriately designed G-quadruplex ligands have played a crucial role in recognizing such structures, promoting telomere damage and therefore favoring cell death [3].

The rapid growth of nanotechnology in the last decades has allowed the design of a plethora of nanostructures for medicine opening up novel and promising alternatives to classical treatments [9]. In this regard, the usage of liposomes, niosomes and polymers has generated great interest in therapy due to their nanometric size, easiness of production, storage, as well as improvements in the transport of certain small therapeutic drugs and macromolecules [10,11,12]. In addition, the preparation of second and third generation of such nanovehicles has introduced novel features such as the enhancement of half-lives in bloodstream and effectiveness by tailoring with specific ligands, antibodies or aptamers to favor targeted delivery [13,14].

While liposomes and other colloidal formulations have been widely used in nanomedicine with success, other strategies involving nanometric sizes have also become promising tools in therapy [15]. DNA nanotechnology represents an emerging approach that relied on employing synthetic building blocks with the aim of creating nanoscale architectures precisely with various applications, including biosensing, genomics, proteomics, drug delivery and gene therapy, among others [9,16,17,18,19]. Multiple self-assembling nanoparticles and bio-inspired DNA materials exhibiting different shapes (e.g., cubes, octahedra, icosahedra, and tetrahedra), including DNA origami have been properly engineered in order to increase their selectivity, efficiency, prolong stability and reduce the side effects of classical therapeutic approaches [18,20]. The construction of this variety of nanostructures, acting as nanocarriers, has made important contributions to developing strategies in many in vitro and in vivo models with the aim to deliver small drug therapeutics (e.g., Doxorubicin [21], 5-fluoro-2-deoxyuridine (5-FdU) [18], among others), RNAi therapeutics [20] or CpG oligonucleotides [22].

**Figure 1:**
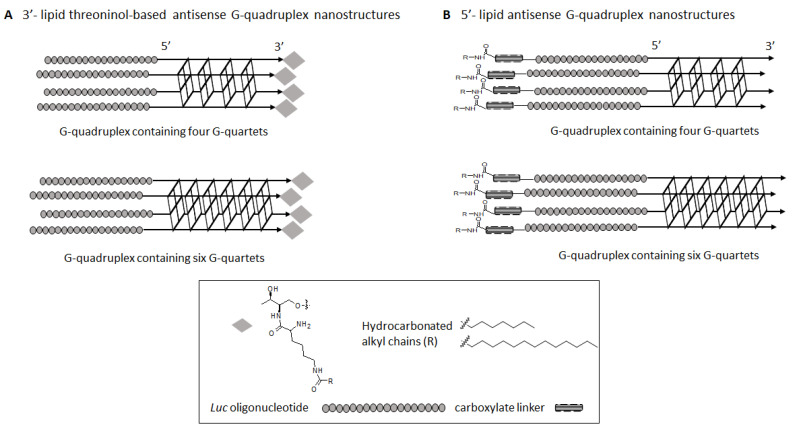
Two series of G-quadruplex forming oligonucleotide conjugates containing four and six G-quartets were engineered: (**A**) a threoninol-based building block modified with two lipids of different length (C8 and C14) was introduced covalently at the 3′-termini of a G-rich oligonucleotide sequence and (**B**) two hydrocarbon lipids of different length (C8 and C14) were introduced at the 5′-termini of a G-rich oligonucleotide sequence using a carboxylate linker. An 18-mer phosphorothioate (*Luc*) oligonucleotide was also attached at the 5′-termini of the G-rich sequence oligonucleotide.

Aptamers, with many of them exhibiting DNA or RNA G-quadruplex motifs, have been used as diagnostic platforms and targeting ligands to facilitate drug delivery processes [23,24]. Other simpler G-quadruplex forming oligonucleotides like *Tetrahymena* telomere dTG_4_T, which is able to self-assemble to give parallel tetramolecular structures, have been used as a G-quadruplex model in many applications involving binding processes and the identification of new ligands for anticancer therapy [25]. Interestingly, modifications of this G-quadruplex have also been reported by introducing carbohydrates [26] and cationic amino acids [27] using covalent strategies or the formation of supramolecular dendrimers. The use of [d(TG_4_T)]_4_ quadruplex as a nanostructure to transport biomolecules has been scarcely investigated [27]. Our group recently proposed the use of positively charged nanoconstructs based on the *Tetrahymena* telomere sequence dTG_4_T with the aim to favor the delivery of an antisense (AS) oligonucleotide in human cancer cells. To do so, our strategy relied on chemically introducing a series of cationic amino acids at the 3′- or 5′-termini of the AS oligonucleotide. This synthetic strategy allowed us to obtain stable parallel cationic G-tetrads that were able to transport and internalize AS oligonucleotides inside cells and therefore inhibit the production of the luciferase reporter protein [27]. In addition, lipid-oligonucleotide conjugates forming G-quadruplex (lipoquads) have been described by some of us to be potent antiviral compounds against human immunodeficiency virus (HIV-1, HIV-2) [28] and hepatitis C virus (HCV) by inhibiting viral entry [28,29]. These antiviral oligonucleotides formed parallel tetramolecular structures with 6 G’s and carried cholesterol or linoleyl moieties at the 3′-end [28,29]. In order to gain further insight into the application of such supramolecular scaffolds in gene silencing, we have extended these studies by modifying the 3′- or the 5′-termini of such G-quadruplex nanostructure with two saturated lipids of different lengths. To do so, two synthetic methodologies involving solution and solid-phase strategies were accomplished to afford a series of hydrophobic series containing two supramolecular G-tetrad scaffolds like [d(TG_4_T)]_4_ and [dTG_6_T]_4_, as well as an 18-mer AS oligonucleotide (Figure 1). In this article, the synthesis and biophysical characterization of such hybrid nanostructures, including their effect on cellular viability and use in antisense therapy, were assessed.

## 2. Results and Discussion

Chemical modification of oligonucleotides with hydrophobic residues represents a useful strategy to modulate the intrinsic properties of nucleic acids [30]. Thus, cationic lipids [31,32], neutral lipids [33,34] and fatty acids [35] have been attached covalently either at the 3′- or 5′-ends in order to improve the cellular uptake, stability and pharmacokinetic properties of AS oligonucleotides, small interference RNAs (siRNAs) and microRNAs (miRNAs) [36]. In this regard, very promising results involving lipid oligonucleotide conjugates (LOCs) have been obtained in vivo, leading to successful results when we launched clinical trials [37].

Recently, some research groups have synthesized modified lipid G-quadruplexes showing their similarities to lipid-based micelles in size and proving their efficiency as supramolecular scaffolds to promote cargo release [38,39,40]. Inspired by these results and by our previous findings based on delivering ASOs using G-quadruplex nanostructures modified with cationic amino acids [27], we envisaged engineering two series of G-quadruplex forming oligonucleotides varying the number of guanosines in the oligonucleotide chain (TG_4_T and TG_6_T) and containing two saturated lipids of different length (C8 and C14), which were introduced at either the 3′- or 5′-termini of the G-rich oligonucleotide chain. To do so, two synthetic strategies were accomplished: (i) a threoninol derivative [41] bearing the corresponding hydrophobic residues was obtained using solution-phase strategies in order to modify the 3′-termini and (ii) CPG solid supports were properly modified with a carboxylate group using solid-phase strategies that enabled the introduction of the two selected amino lipids (octylamine and tetradecylamine) at the 5′-end.

### 2.1. Synthesis of Lipid Threoninol Derivatives and Preparation of Lipid Oligonucleotide Conjugates (LOCs) with G-Rich Sequences

**Figure 2:**
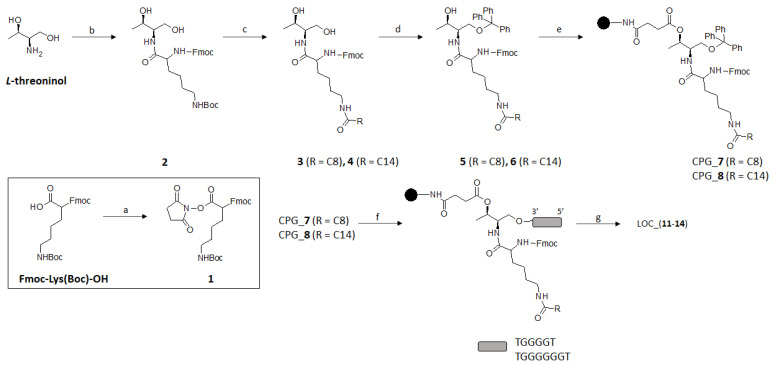
Synthetic route devised to obtain CPG solid supports modified with octyl and tetradecyl alkyl chains and lipid oligonucleotide conjugates (LOCs). Reagents and conditions: a. NHS, EDCI, DCM, r.t.; b. (**1**), DMF, r.t., overnight; c. (i) DCM:TFA 10%, r.t., 30 min; (ii) AcONa 50% *w/v*, THF:H_2_O (1:1), RCOCl (R = octyl chloride or tetradecanoyl chloride, r.t., overnight; d. TrCl, DIPEA, pyridine, 45 °C, overnight; e. CPG functionalization; f. aq. NH_3_ (32%), 55 °C, overnight; g. (i) HPLC purification; (ii) AcOH 80%, 30 min, r.t.; (iii) NAP-10.

We envisaged the chemical modification of our starting material with two hydrocarbon alkyl chains (C8 and C14) that could be tackled previously by activating Fmoc-Lys(Boc)-OH with *N*-hydroxysuccinimide (NHS) in standard conditions affording the intermediate (**1**) (Figure 2). This activated molecule proceeded smoothly when reacted with *L*-threoninol, yielding the expected protected threoninol derivative (**2**) in good yield (75%). Subsequent Boc deprotection under acid conditions (10% TFA in CH_2_Cl_2_) afforded a rapid access to nucleophilic addition of the amine derivative with the corresponding acyl chlorides of different lengths. To avoid undesirable *O*-acetylated derivatives, we pursued a synthetic approach favoring the formation of *N*-acetylated compounds following the Schotten-Baumann conditions (50% *w/v* aq NaOAc in THF) [42]. This biphasic strategy led to the formation of threoninol derivatives (**3**) and (**4**), modified with the C8 and C14 saturated alkyl chains in good yields (97 and 78%, respectively). The last step, prior to Controlled Pore Glass (CPG) functionalization, was relied on protecting the threoninol’s primary alcohol with a trityl (Tr) protecting group. This bulky protecting group is essential to start up automatic DNA synthesis and therefore to synthesize the corresponding oligonucleotide sequences. We selected the Tr group instead of the standard DMT group because DMT derivatives were not stable enough in silica gel. Isolation of the resultant trityl threoninol derivatives (**5**) and (**6**) were challenging due to their hydrophobicity, affording overall low yields after purification by flash chromatography (24 and 53% for **5** and **6**, respectively). Finally, CPG functionalization took place efficiently with the elaboration of the corresponding hemisuccinate derivatives obtained from the opening of succinic anhydride mediated by DMAP and alcohols (**5**) and (**6**) in the solution-phase. Finally, a coupling reaction between the CPG beads’ amine groups and hemisuccinate derivatives [43] allowed us to functionalize the polymer supports and thus afforded the corresponding functionalized CPG-**7** (37.6 μmol·gr^−1^) and CPG-**8** (23.0 μmol·gr^−1^). Taking both CPG beads in our hands, it allowed us to initiate the solid-phase oligonucleotide synthesis and thus modifying the 3′-termini of two G-rich proof-of-concept sequences such as d(TG_4_T) and d(TG_6_T). Finally, the corresponding lipid oligonucleotide conjugates were detached from the solid support under ammonia treatment at 55 °C affording four threoninol-based LOCs (**11–14**), which were purified according to DMT-*on* protocols and characterized by MALDI-TOF spectrometry (Table 1).

### 2.2. Preparation of Lipid Oligonucleotide Conjugates (LOC) Hybrids Containing the Luc Sequence

The appropriate design and selection of antisense oligonucleotide sequences is key to achieve successful results in antisense therapy. We decided to use an 18-mer antisense oligonucleotide of sequence 5-CGTTTCCTTTGTTCTGGA-3 (*Luc*) as a model [44] to evaluate the feasibility of our nanoconstructs to bind to the *Renilla* luciferase mRNA sequence specifically.

**Figure 3:**
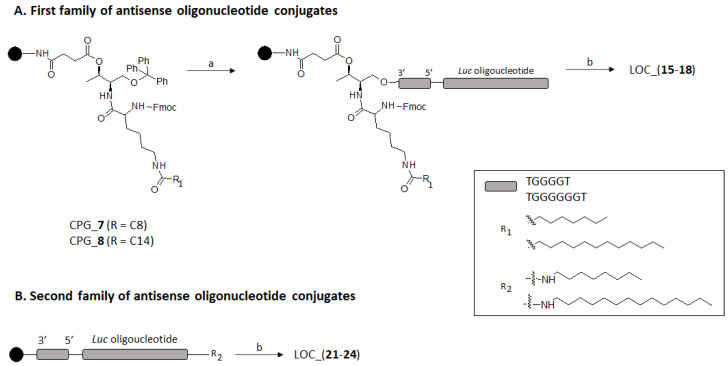
Preparation of (i) LOC containing threoninol-based hydrocarbon alkyl chains and a *Luc* oligonucleotide at the 3′- and 5′-termini of a G-rich oligonucleotide sequence (TG_4_T and TG_6_T), respectively (Group **A**); (ii) LOC containing a *Luc* oligonucleotide at the 5′-termini of a G-rich oligonucleotide sequence (TG_4_T and TG_6_T) and two hydrocarbon alkyl chains at the 5′-termini of a *Luc* oligonucleotide (Group **B**). Reagents and conditions: a. DNA synthesis and amide formation (5′-modification only); b. (i) aq. NH_3_ (32%), 55 °C, overnight; (ii) HPLC purification; (iii) AcOH 80%, 30 min, r.t.; (iv) NAP-10.

Two LOC series containing two distinct G-rich scaffolds [d(TG_4_T) and d(TG_6_T)] were prepared depending on the position of the lipid modification (Figure 3). Thus, having CPG-**7** and CPG-**8** in our hands, the first set of phosphorothioate oligonucleotide conjugates containing a lipid modification (either octyl, C8 or tetradecyl, C14) and the *Luc* sequence at the 5′-end, respectively, were obtained (**15–18**) in accordance with standard DNA synthesis protocols. Alternatively, a second family of phosphorothioate lipid oligonucleotide antisense conjugates containing the same G-rich scaffolds, as described before, was devised by introducing two hydrophobic amino lipid tails with the same length (C8 and C14), as introduced above, but at the 5′-termini of the *Luc* sequence. To do so, a protected carboxylate modifier phosphoramidite acting as a spacer was introduced in order to modify the 5′-antisense sequence termini and assist the final conjugation between the two selected amino lipids and the DNA sequence on solid-phase. After manually removing the 2-chlorotrityl protecting group under acid conditions (2% TCA), the amide-bond formation took place prior to activating the carboxylic group with a mixture of PyBOP and HOBt (1:1) in an organic solvent (DMF) [45]. Finally, the resultant 5′-oligonucleotide conjugates containing octylamine and tetradecylamine (**21–24**) were detached from CPG solid supports under standard basic conditions and the yield of the reaction was analyzed by HPLC, which ranged from 60% to 80%. All LOC containing both d(TG_4_T) and d(TG_6_T) building blocks (LOC_(**15–18**) and LOC_(**21–24**)) were isolated according to well established protocols, purified by semi-preparative HPLC and characterized by MALDI-TOF mass spectrometry (Table 1).

### 2.3. G-Quadruplex Formation and Biophysical Characterization

**Figure 4:**
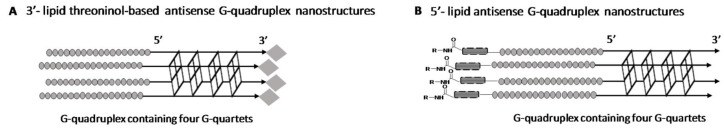
G-quadruplex forming oligonucleotide conjugates prepared in this study. (**A**). Lipid threoninol-based modifications were introduced at the 3′-termini of the G-rich sequence and (**B**). Lipid amino lipids were introduced, modifying the 5′-terimni of the G-rich sequence.

**Figure 5:**
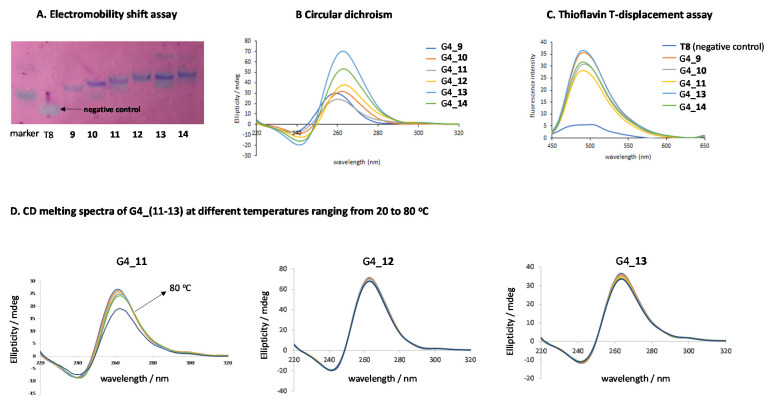
Native PAGE analysis, circular dicrhoism (CD), CD melting spectra, and Thioflavin T (ThT)-displacement assay of G-quadruplex forming oligonucleotides G4_(**9-14**). (**A**) Native 20% polyacrylamide gel electrophoresis analysis of G-forming oligonucleotide conjugates (**9-14**) in 1X PBS supplemented with 100 mM KCl. A mixture of bromophenol blue and xylene cyanol was used as a marker. T8 is an oligonucleotide containing 8 thymidines that was used as a negative control as it is unable to form a quadruplex and indicate the position of a single-stranded 8-mer. (**B**) CD spectra measured at 20 °C showing positive and negative bands at 264 and 240 nm, respectively. This confirms the presence of a parallel G-quadruplex construct. G-constructs were obtained in 10 mM sodium cacodylate, 110 mM NaCl buffer; (**C**) ThT displacement assay shows the emitted fluorescence of ThT probe when bounded to G-quadruplex structures. A T-rich oligonucleotide (T8) was used as a negative control; (**D**) CD melting spectra at several temperatures ranging from 20 to 80 °C with a heating rate of 1.0 °C min^−1^. All conjugates showed remarkable stabilities except, for G4_**11,** which had a band intensity that was slightly reduced.

G-quadruplex (G4) forming oligonucleotide conjugates were prepared and characterized (Figure 4 and Figure 5) from their lipid oligonucleotide counterparts (**11–24**) in the presence of two buffer solutions: 10 mM sodium cacodylate buffer (pH 7.2) supplemented with 0.11 M NaCl [46] and 10 mM phosphate buffer solution (pH 7.4). In this regard, sodium cacodylate buffer was used to dissolve G-rich oligonucleotide conjugates (**11–14**), and upon the addition of NaCl, favored their self-assembling to form the expected G4 nanostructures G4_(**11–14**) bearing four and six G-quartets, respectively. The quadruplex formation of unmodified and 3′-lipid constructs (**9–14**) were visualized after running and staining a native polyacrylamide gel in 1X TBE supplemented with 100 mM KCl. As illustrated in Figure 5A, the presence of the lipids (C8 and C14) at the 3′-termini did affect either the formation or the stability of the aforementioned quadruplexes obtaining the expected nanostructures, exclusively. Octathymidylate (T8) oligonucleotide was used as a negative control and for this reason the gel was stained with “stains-all”. To further study the contribution of hydrophobic residues on the G4 telomeric stability, circular dichroism, CD melting analyses and the Thioflavin T-displacement assay were carried out taking unmodified quadruplexes G4_**9** and G4_**10** as controls. The unmodified tetramers [d(TG_4_T)]_4_ and [d(TG_6_T)]_4_ are four-stranded parallel structures that typically display two bands; one positive at 264 nm and another negative at 240 nm according to standard CD spectra [47]. This behavior was also observed in our G4-conjugates (**11–14**) making clear that hydrophobic 3′-threoninol derivatives did not alter G4 formation at room temperature, as observed in other modified G4 nanostructures [27] (Figure 5B). Additionally, Thioflavin T-displacement assay [48,49] was used to confirm the presence of the G-quadruplex structures in **9–14** using a single-stranded T-rich oligonucleotide (T8) as a negative control. Thus, equimolecular amounts of ThT acting as a fluorescent dye, unmodified G-quadruplex-forming oligonucleotides (**9**, **10**), and G-quadruplex forming oligonucleotide conjugates **(11**, **13**, **14**) were mixed separately with increasing concentrations of ThT (Figure 5C). As expected, ThT probe emitted fluorescence when recognized and bounded to our pre-formed G4-constructs thus confirming the presence of lipids attached at the 3′-termini did not disturb the G-quadruplex formation.

Thermal denaturation experiments of modified quadruplexes (**11–14**) were assessed in 10 mM sodium cacodylate, 110 mM NaCl buffer recording the ellipticity at 263 nm as a function of the temperature from 20 to 80 °C (Figure 5D). Petraccone et al. already observed that a greater number of G-quartets present in telomeric sequences produced an increase of their thermal stability [47]. This behavior was also observed here, when the apparent melting temperatures (T_1/2_) of the prepared tetramers (**11–14**) were determined. It is worth mentioning that G4 tetramers are not usually found in a thermodynamic equilibrium during unfolding processes as their association/dissociation rates are usually slow [50]. In this regard, when the unmodified tetramer G4_**9** was heated up at 80 °C with a heating rate of 1.0 °C min^−1^, negative and positive bands at 240 and 263 nm, respectively, reduced in intensity, recording an apparent melting temperature of 59.2 °C [27]. Interestingly, we found the presence of hydrophobic threoninol-based derivatives in G4 constructs containing their four and six G-quartets modified at the 3′-termini had a meaningful impact on the mechanism that governs G4 dissociation processes. In this sense, while TG_4_T had a clear melting profile at around 59 °C [27], the 3′-octyl derivative TG_4_T_*C8* (**11**) showed only a minor decrease in the band intensity at 80 °C. In the other 3′-lipid G-quadruplex studied (TG_4_T_*C14* (**12**), TG_6_T_*C8* (**13**) and TG_6_T_*C14* (**14**),) we did not observe any changes in the CD spectra up to 80 °C (Figure 5C and Table 1), indicating that the G-quadruplex formed was very stable.

Naively, we tried to confirm the formation of parallel quadruplex structures containing *Luc* oligonucleotide by CD. Despite showing the negative band at 240 nm, the addition of 18 bases antisense sequence did not allow for the valuable CD spectral data as the additional 18 bases present CD signals near the positive band of parallel quadruplex at 264 nm interfering the analysis of the bands. For this reason, the formation of the G-quadruplex in the oligonucleotides carrying the luciferase sequence was visualized in native PAGE gels (Figure S1, Supplementary Information). Curiously, we observed that G-rich conjugates containing *Luc* oligonucleotide displayed a different trend when lipids were introduced either at 3′- or 5′-termini. While antisense G-quadruplex conjugates containing hydrophobic residues at the 3′-termini showed the G-quadruplex secondary structure practically was unchanged in most cases, this was the opposite in the case of lipid modifications (C8 and C14) introduced at the 5′-termini. Unexpectedly, native PAGE gels showed mixtures of two bands that were assigned for the mobility to the monomeric form and the G-quadruplex after being stained with SYBR green (Figure S1, Supplementary Information). As a general trend, the presence of lipids at the 5′-end disrupted G-quadruplex formation while most of the 3′-modified G-rich sequences maintained the band assigned to G-quadruplex as the major band. The unmodified G-rich sequences **19** (*Luc*-TG_4_T) and **20** (*Luc*-TG_6_T) showed a different behavior. The major band of *Luc*-TG_4_T (**19**) was assigned to monomer while the major band of oligonucleotide *Luc*-TG_6_T (**20**) was assigned to quadruplex. Likewise, ThT assays were also assessed to further confirm the results observed in the PAGE native gel. Following the same experimental protocols carried out in the case of G-quadruplex structures described above, representative antisense G-quadruplex-forming oligonucleotide conjugates containing both lipid modifications and *Luc* oligonucleotide G4_(**15**, **16**) and controls G4_(**9**, **10** and **19**) and *Luc* oligonucleotide alone were mixed separately with increasing concentrations of ThT (see Supplementary Material). Surprisingly, ThT dye bounded to our single-stranded *Luc* phosphorothioate oligonucleotide that emitted significant fluorescence intensities (Supplementary Information), but slightly less than our pre-formed antisense G4-quadruplex conjugates selected in this study, except for G4_**19,** which obtained maximum fluorescence intensity values. The affinity constants and G-quadruplexes are also listed in Table S1 (see Figure S3; Supplementary Information).

### 2.4. In Vitro Transfection Studies

After the biophysical characterization, G-constructs containing a phosphorothioate oligonucleotide (*Luc*) as an additional pendent group were engineered in order to make these nanostructures as potential vehicles to be used in antisense therapy.

**Figure 6:**
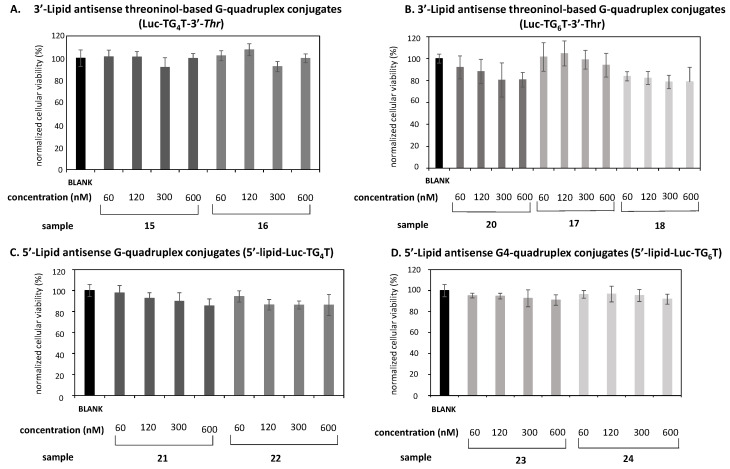
Cytotoxicity analysis of antisense conjugates containing four Gs (**A**,**C**) and six Gs (**B**,**D**). A lipid threoninol-based modification at the 3′-termini were introduced (**A**,**B**); a lipid modification (C8, C14) was introduced at the 5′-termini (**C**,**D**). Four concentrations of antisense conjugates, ranging from 60 nM to 600 nM, were used. All antisense conjugates were incubated up to 24 h at 37 °C. Data were means ±SD of three independent experiments.

Some G-quadruplexes, particularly those pre-formed in the presence of potassium, have proved their efficacy as proliferation inhibitors inducing apoptosis in HeLa cervical carcinoma cells at 10 µM as a final concentration in a single dose [51]. Before carrying out gene silencing studies, the effect of our modified G-rich oligonucleotides on cellular viability was first assessed. Cytotoxicity analysis was studied using the MTT colorimetric assay [52]. As shown in Figure 6, two sets of antisense G-rich oligonucleotides were tested, depending on the number of G-tetrads these conjugates were made up of and the position of the lipid modification (3′- or 5′-) (**15–18** and **21–24**). Two unmodified controls containing exclusively *Luc* oligonucleotide and four Gs [27] and six Gs (**20**) were also used. Four increasing concentrations of the corresponding antisense conjugates were studied (60, 120, 300 and 600 nM), which did not have any influence on the proliferation process of HeLa cells after 24 h-incubation. As a consequence, cellular viabilities ranging from 80% and 100% were obtained when compared to non-treated cells (*Blank*), implying that these G-rich oligonucleotides could be used in subsequent gene silencing studies. In addition, the potential toxicity of these compounds on a non-cancerous cell line (HEK293) was assessed using the same oligonucleotide concentrations, as described above (60, 120, 300 and 600 nM). The results showed cellular viabilities ranging from 80–100%, confirming that our compounds were non-toxic to HEK293 cells (Figure S2, Supplementary Information).

**Figure 7:**
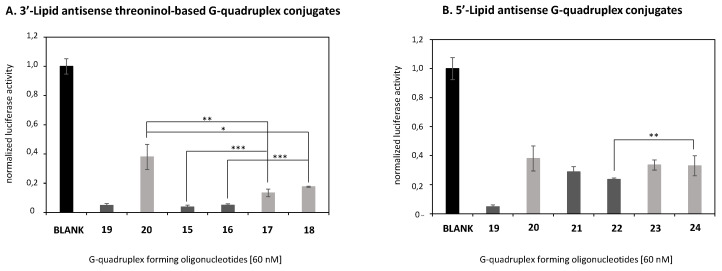
Gene transfection studies targeting *Renilla* luciferase mRNA in the presence of Lipofectamine 2000 involving antisense G-quadruplex forming oligonucleotides **15-24** at 60 nM. Two series of conjugates containing four and six guanines were studied. (**A**) antisense G-rich modified with lipid threoninol-based building blocks at the 3′-termini and (**B**) lipids were conjugated by modifying the 5′-termini of the G-rich oligonucleotide. Two alkyl chains were selected according to their length (C8 and C14). Two unmodified G-rich oligonucleotides were used as controls (**19** and **20**). Data were means ±SD of three to six independent experiments. A regular two-way ANOVA variance analysis combined with Bonferroni post-test for multiple comparisons was evaluated (* *p* < 0.05, ** *p* < 0.01 and *** *p* < 0.001).

Having established that our G-rich antisense oligonucleotides **17–24** did not compromise the cellular viability of HeLa cells, the ability of such oligonucleotides to transfect cells and knockdown *Renilla* luciferase mRNA was studied. Transfection experiments in the presence of lipofectamine and without using this commercially available cationic lipid were carried out. Recently, our group confirmed that G-quadruplex constructs, modified with a series of cationic amino acids at the 3′-termini, were able to reduce luciferase protein expression thereby confirming these nanostructures did not disrupt any AS oligonucleotide-mediated mechanisms [27]. To confirm our G-rich oligonucleotides **17–24**, which did not affect the aforementioned antisense machinery, they were mixed with lipofectamine 2000 and luciferase inhibition activities were evaluated after 24 h-incubation as displayed in Figure 7. Interestingly, when analyzing the silencing activity of the oligonucleotides formed by *Luc*-TG_4_T (**19**) and *Luc*-TG_6_T (**20**) without any modification at either 3′ or 5′, we found a clear decrease of the silencing activity when moving from a 4-Gs to a 6-Gs (91% and 62%, respectively) (Figure 7A). Curiously, this tendency is contrary to the stability of the potential quadruplex as the more stable quadruplex (*Luc*-TG_6_T (**20**)) displayed a reduced silencing activity. Thus, as this experiment was carried out with a transfecting agent, the silencing activities exhibited by both antisense oligonucleotides **19** and **20** cannot be explained based on their cellular uptake efficiencies, but other factors are needed to explain the lower antisense activity of the 6-Gs oligonucleotide (**20**). For example, differences on the endosomal escape or in their efficiency in the binding process to target mRNA might also be taken into account. Next, we observed that the addition of the lipid at the 3′- improved the silencing activity on the 4-Gs oligonucleotide (96% *Luc*-TG_4_T_*C8* (**15**) and 95% *Luc*-TG_4_T_*C14* (**16**) when compared to the 6-Gs oligonucleotide counterparts (87% *Luc*-TG_6_T_*C8* (**17**) and 82% *Luc*-TG_6_T_*C14* (**18**)) (^***^p < 0.001). These data might give support to a more efficient endosomal escape rather than an increased binding affinity to target mRNA, but more experiments need to be done to confirm this hypothesis. Additionally, we observed that unmodified *Luc*-TG_6_T (**20**) was less efficient in silencing luciferase expression than their counterparts modified with lipid threoninol moieties *Luc*-TG_6_T_*C8* (**17**) and *Luc*-TG_6_T_*C14* (**18**) (62% versus 87% and 82%, respectively) (** *p* < 0.01 and * *p* < 0.05). These results may suggest the role of alkyl chains as well as the presence of threoninol moieties when conjugated covalently with oligonucleotides in order to favor endosomal escape or an increased binding affinity [33,53].

The silencing efficiency of antisense G-rich oligonucleotides modified with lipids at the 5′-termini (C8_*Luc*-TG_4_T (**21**), *C14*_*Luc*-TG_4_T (**22**), C8_*Luc*-TG_6_T (**23**) and *C14*_*Luc*-TG_6_T (**24**)) was also studied (Figure 7B). The results showed lower silencing activities of 5′-lipid conjugates when compared with their quadruplex counterparts modified at the 3′-termini (71%, 76%, 66% and 67% versus 96%, 95%, 87% and 82%, respectively). This difference in silencing activities might be explained by differences in the degradation process mediated by nucleases including distinct quadruplex stabilities observed in SDS-PAGE gels. Thus, when the lipid is at the 3′-end, oligonucleotide conjugates contain nuclease resistant moieties at both ends (3′-lipid; 5′-phosphorothioate), while when the lipid is added at the 5′-position, we have both nuclease resistance moieties in line leaving the 3′-end of the oligonucleotide unprotected to exonucleases. Thus, it may be the 5′-lipid modified oligonucleotides that are prone to be degraded more rapidly if compared to the 3′-lipid modified oligonucleotides. Another potential reason may also be the low efficiency on G-quadruplex formation observed for the 5′-lipid modified oligonucleotides (**21–24**). This may be another factor to increase nuclease degradation.

**Figure 8:**
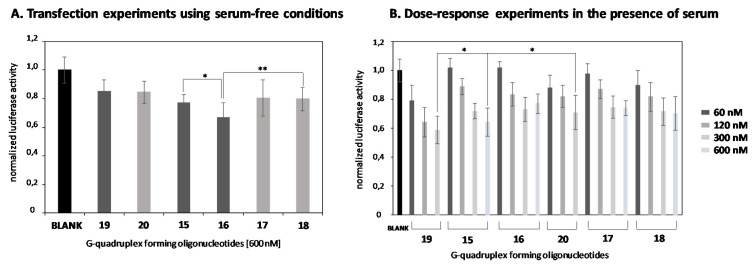
Gene transfection studies targeting *Renilla* luciferase mRNA without using lipofectamine in serum-free conditions (**A**). Modified and unmodified antisense conjugates (**15–18**) and (**19**, **20**) were used at 600 nM. (**B**) Dose-response transfection experiments using several concentrations (60, 120, 300 and 600 nM) in the absence of lipofectamine. G4_(**19**, **20**) were used as controls. Data were means ±SD of three to six independent experiments. A regular two-way ANOVA variance analysis, combined with Bonferroni post-test for multiple comparisons, was evaluated (* *p* < 0.05 and ** *p* < 0.01).

According to the silencing activities displayed above, we selected oligonucleotides **15–18** as the most potent antisense constructs for further experiments. Gymnotic transfection experiments using serum-free conditions remarkably reduced *Renilla* mRNA knockdown efficiencies when **15–18** constructs were used in the absence of lipofectamine (Figure 8A). Interestingly, silencing activities followed the same trend as observed in Figure 7A, in which constructs containing four Gs exhibited slightly better inhibition activities (23% and 33% for **15** and **16**, respectively) than G-rich constructs containing six Gs (19% and 20% for **17** and **18**, respectively) at 600 nM. These outcomes revealed that long alkyl chains tended to interact more efficiently with the cellular membrane [54] by the inclusion of **16** and to a lesser extent **15**- into phospholipid bilayers promoting their destabilization and therefore leading to significant inhibition activities when compared to **15** and **18** (* *p* < 0.05 and ** *p* < 0.01, respectively). As expected, unmodified antisense G-rich constructs (**19** and **20**) were not able to inhibit luciferase in the same fashion that the modified nanostructures did (15% and 16%, respectively).

Once the transfection process of conjugates was studied in serum-free conditions, dose-response experiments were then assessed to evaluate the effect of serum proteins on oligonucleotide delivery using four concentrations (60, 120, 300 and 600 nM). As shown in Figure 8B, all modified G-rich constructs (**15–18**), as well as unmodified G-rich counterparts (**19** and **20**), were able to knockdown *Renilla* luciferase mRNA in a dose-response manner but with distinct effectiveness. Interestingly, the presence of serum proteins did not disrupt the transfection process of both unmodified and modified G-rich conjugates. According to Figure 7B, it is worth mentioning that oligonucleotide **15,** which was made up of four potential G-quartets and a saturated alkyl chain of 8 atoms of carbons in length, showed the best silencing activity (36% of inhibition) of the two G-rich families, though these results were not considered statistically significant. Curiously, G-rich constructs modified with the long hydrophobic C14 alky chain (**16**) did not afford a greater increase in knocking down *Renilla* luciferase expression than the one observed in serum-free conditions (22% versus 33%, respectively). This behavior underscores the role of serum proteins in promoting the transport of such nanostructures within cells. In this regard, experiments showed that certain saturated lipids tended to interact efficiently to albumin, which is the major component of serum, facilitating not only the formation of lipid-binding proteins complex, but also the delivery of oligonucleotide conjugates [55,56]. However, it was noted that this binding process was not as effective in the case of long alkyl chain moieties [57]. Consequently, this produced lack of effectiveness in the interaction of such lipids to albumin and thereby could reduce the ability of G-rich sequence **16** to promote cell entry in some way.

Remarkably, unmodified G-rich sequence **19** showed better and statistically significant silencing values than the unmodified G-rich sequence **16** containing six Gs (* *p* < 0.05), as well as modified G-rich sequence **15** counterparts at high concentrations (300 nM) (* *p* < 0.05). This effect was already observed previously [27] and revealed other parameters including receptor-mediated processes might be involved in promoting silencing of *Renilla* luciferase.

### 2.5. Cellular Uptake Studies

A fluorescently labeled F_G-rich_**15** conjugate (**25**) and a F_T-rich (**26**) conjugate were prepared to carry out internalization studies in HeLa expressing nucleolin and human embryonic kidney (HEK293) cells. To do so, a phosphoramidite-based approach was used to introduce a fluorescein dye at the 5′-termini of phosphorothioate *Luc* oligonucleotide. The ability of such constructs to transfect HeLa and HEK293 cell lines was evaluated by flow cytometry analysis.

**Figure 9:**
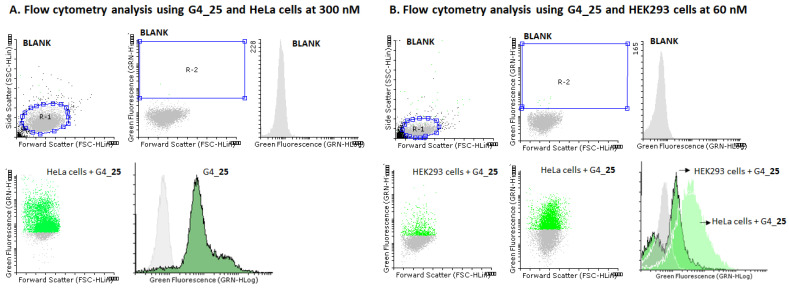
Flow cytometry analysis involving the effect of the G-rich sequence **25** when transfecting HeLa (**A**) and HEK293 cells (**B**). (**A**) Transfections were carried using G-rich sequence **25** at 300 nM. First row: non-fluorescent labelled cell populations (Blank); selected region R-2 (center) and histogram of non-fluorescent cells (right); second row: forward scatter dot plot (left) and histogram (right) of G-rich sequence **25** in the presence of HeLa cells at 300 nM. (**B**) Transfections were carried using G-rich sequence **25** at 60 nM. First row: non-fluorescent labelled cell populations (Blank); selected region R-2 (center) and histogram of non-fluorescent cells (right); second row: forward scatter dot plot (left) using G-rich sequence **25** and HEK293 cells; forward scatter dot plot (middle) using G-rich sequence **25** and HeLa cells and combined histogram (right) of G-rich sequence **25** in the presence of HeLa and HEK293 cells at 60 nM. The Flowing Software 2.5.1 was used to measure the relationship between untreated cells and positive cell populations.

Figure 9A illustrates a histogram showing a remarkable displacement of fluorescently labeled HeLa cell populations (84%) when compared to non-treated cells after having been used in the G-rich sequence **25** at 300 nM. It was noted that the displacement of fluorescence HeLa cells was dependent on the concentration used, showing a smaller number of labelled cells at 60 nM (69%) (Figure 9B). In this regard, flow cytometry experiments using the same concentration (60 nM) of the G-rich **25** construct was carried out in the presence of HEK293 cells (Figure 8B). Interestingly, the number of labelled HEK293 cells obtained after transfection was lower than in Figure 8A and dropped to 16%, which might be an indication of the preference of G-rich sequence constructs for nucleolin receptors expressed in the surface of HeLa cells. To evaluate the affinity of G-rich sequence **25** on transfection, conjugate **26** containing the same threoninol modifications at the 3′-termini and a *Luc* oligonucleotide and fluorescein at 5′-termini of a T-rich sequence (TTTTTT) was prepared. Surprisingly conjugate **26** was able to impart cellular uptake with similar efficiencies and no statistically significant differences were found in HeLa cells when compared to G4_**25** at two concentrations (60 and 300 nM) (Figures S3 and S4, Supplementary Material). Despite molecular mechanisms involving oligonucleotide uptake are not fully understood in some cases, the lack of selectivity we observed between G-rich and the T-rich conjugates might be justified due to the presence of phosphorothioate *Luc* oligonucleotide in both constructs. In this regard, phosphorothioate oligonucleotides have shown to be prone to interact indistinctly to several binding proteins like albumin or nucleolin, among others, which might affect and compromise both the potency and subcellular location of such oligonucleotide conjugates in the cell culture line used in this study [58,59,60].

### 2.6. Comparison of the Luciferase Inhibitory Properties of Antisense Oligonucleotides Carrying TG4T or T6 Sequences

**Figure 10:**
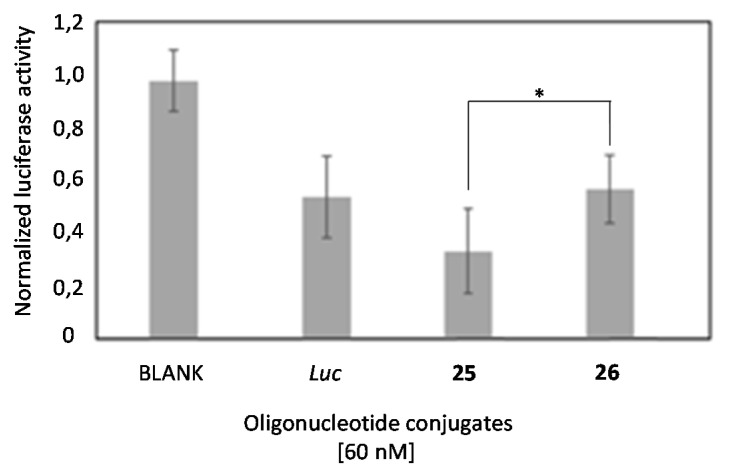
Gene transfection studies targeting *Renilla* luciferase mRNA in the presence of Lipofectamine 2000 involving antisense G-rich oligonucleotide **25** and two oligonucleotide controls: the T-rich **26** and the *luc*-sequence (ASO) at 60 nM. Two unmodified G-rich oligonucleotides were used as controls (**19** and **20**). Data were means ±SD of three to six independent experiments. A regular two-way ANOVA variance analysis combined with Bonferroni post-test for multiple comparisons was evaluated (* *p* < 0.05).

The small differences observed on the cellular uptake of G-rich (**25**) and T-rich (**26**) oligonucleotides prompted us to analyze, side by side, if there was a difference in the luciferase inhibitory properties of these oligonucleotides. Figure 10 shows the luciferase activity of oligonucleotides **25** and **26** using lipofectamine 2000 as a transfecting agent in HeLa cells after 24 h of treatment. Here, we used lipofectamine 2000 to equally internalize compounds **25** (G-rich oligonucleotide), **26** (T-rich oligonucleotide) and the antisense luciferase oligonucleotide (*Luc*) at 60 nM. As illustrated in Figure 9, greater and significant inhibition of the G-rich oligonucleotide **25** was achieved if compared to the T-rich sequence **26** (* *p* < 0.05) and *Luc* oligonucleotide, as a control.

**Figure 11:**
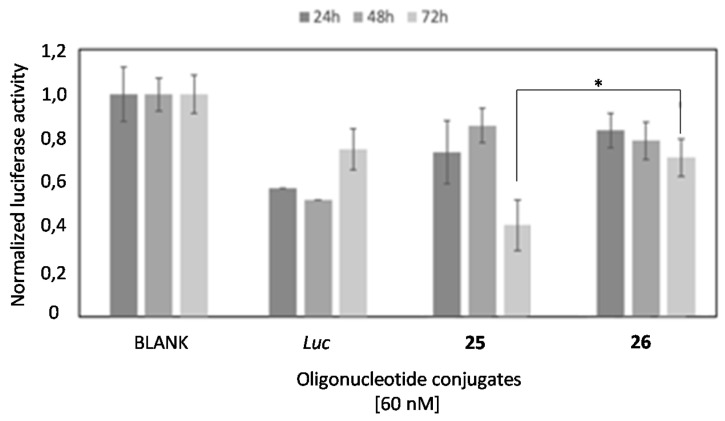
Gene transfection studies targeting *Renilla* luciferase mRNA without using transfecting reagents involving antisense G-rich oligonucleotide **25** and two control oligonucleotides such as the T-rich **26** and the *luc*-sequence (ASO) at 60 nM. Data were means ±SD of three to six independent experiments. A regular two-way ANOVA variance analysis combined with Bonferroni post-test for multiple comparisons was evaluated (* *p* < 0.05).

This result shows a clear inhibitory advantage for the G-rich oligonucleotide. This increased efficacy might result from a positive contribution of the G-rich structure in the endosomal escape [61] and/or an increased efficacy of the oligonucleotide in the binding and subsequent RNase H degradation of target luciferase mRNA [62]. Next, we evaluated the long-term silencing properties of these two oligonucleotides (**25** and **26**) and *Luc* oligonucleotide without using a transfecting reagent. Figure 11 shows the luciferase concentration obtained after treatment of HeLa cells with these oligonucleotides (60 nM) after 24, 48 and 72 h.

While the most active oligonucleotide was the standard anti-luciferase oligonucleotide (*Luc*) after 24 h-incubation, it was probably due to its smaller size and was fully protected by a phosphorothioate backbone, and its efficacy decreased at longer times (72 h). However, we observed that the G-rich oligonucleotide **25** became more active after enlarging the incubation time up to 72 h. In this sense, the T-rich oligonucleotide **26** had a similar trend than **25 but** was much less active. Taking all these results together, we can conclude that the presence of the G-rich sequence increases the inhibitory properties of the antisense oligonucleotide but the advantage might be related with a potential improvement of the endosomal escape rather than the predicted increase of cellular uptake. More research needs to be done to confirm this interesting hypothesis.

## 3. Materials and Methods

### 3.1. General Methods and Materials

All chemical reactions were carried out under inert atmosphere using anhydrous solvents and oven-dried glassware. Chemical reactions were stirred magnetically. Chemicals were purchased from Sigma-Aldrich (St. Louis, MO, USA) and used directly without further purification. Analytical thin layer chromatography (TLC) was used to monitor all reactions. TLC plates (Alugram SilG/UV_254_) were visualized either under UV light or by staining with a phosphomolybdic acid solution. Flash chromatography was performed on 0.063–0.2 mm/70–230 mesh SDS silica gel.

NMR spectra were carried out in the Nuclear Magnetic Resonance (NMR) core facility of the Institute for Advanced Chemistry of Catalonia (IQAC CSIC). ^1^H (400 MHz) and ^13^C (proton decoupled, 100 MHz) NMR spectra were obtained using a Varian Mercury spectrometer (Agilent Technologies, Santa Clara, CA, USA) at 25 °C and referenced to residual signal (Tetramethylsilane, TMS; 0 ppm) and deuterated solvents like CDCl_3_ (7.26 ppm and 77.16 ppm) for ^1^H and ^13^C NMR spectra, respectively. ^1^H NMR coupling constant(s) are reported in hertz (Hz) for peak integration, whereas chemical shifts are reported in part per million (ppm). Multiplicity is reported as follows: singlet (s), doublet (d), triplet (t), m (multiplet) and bs (broad signal). Electrospray ionization mass spectroscopy (ESI-MS) and high-resolution (HR) ESI-MS were carried out in the Scientific and Technological Centers of the *Universitat del Barcelona*. ESI-MS and HRESI-MS were performed on a Micromass ZQ instrument (Milford, MA, USA) with a single quadrupole detector coupled to an HPLC and an Agilent 1100 LC/MS-TOF instrument (Santa Clara, CA, USA), respectively.

Oligonucleotide synthesis was carried out in-house on an Applied Biosystem 3700 instrument (Carlsbad, CA, USA) on a 1 µmol scale using the standard manufacturer’s protocol. Standard phosphoramidites (A, C, G and T), solid resins and ancillary reagents were purchased from Applied Biosystems (Carlsbad, CA, USA), Link Technologies (Lanarkshire, Scotland, UK) and/or Glen Research (Sterling, VA, USA). The isobutyryl (^i^Bu) and benzoyl (Bz) groups were used to protect groups from guanosine (G) and the Adenosine (A) and Cytidine’s (C) amino groups, respectively. The coupling efficiency was greater than 95%. Fluorescent oligonucleotides were prepared using a commercially available 6-FAM phosphoramidite, which was purchased from Link Technologies (Lanarkshire, Scotland, UK). A commercially available 5′-carboxylate modifier-CE phosphoramidite (Carboxy-C5) was purchased from Link Technologies (Lanarkshire, Scotland, UK). Both unmodified and modified oligonucleotides were cleaved from the solid support using a concentrated ammonia solution (55 °C, overnight) and desalted through a NAP-10 column by a gel-filtration (Sephadex G-25) (GE Healthcare) (Pittsburgh, PA, USA). All oligonucleotide conjugates were purified following DMT*on*-based protocols (80% AcOH in water; 30 min at room temperature). Unmodified oligonucleotides were deprotected following DMT*off*-based protocols (aq NH_3_ solution (32%), 55 °C, overnight). Purity of crude samples were analyzed by RP-HPLC using a Waters 2695 Separation Module equipped with a Waters 2998 Photodiode Array Detector. Oligonucleotide conjugates were purified using two buffers: 1. Buffer A: 5% ACN in 100 mM triethylammonium acetate (TEAA; pH 7.0) and 2. Buffer B: 70% ACN in 100 mM triethylammonium acetate (TEAA; pH 7.0). Matrix-assisted laser desorption ionization-of-flight (MALDI-TOF) mass spectra was used to identify the correct mass of the modified oligonucleotides. MALDI-TOF mass spectra were recorded on a Voyager-DE^TM^RP spectrometer in negative mode using 2,4,6-trihidroxyacetophenone and ammonium citrate as a matrix and additive, respectively. UV analyses were used to determine the oligonucleotide concentration (unmodified and modified). These analyses were carried out on a Jasco V-650 instrument equipped with a thermoregulated cell holder. Melting temperatures (*T_m_*) were obtained using MATLAB routines (R2009b version; Math-Works, Natick, MA, USA).

A phosphorothioate oligonucleotide sequence [44] [d(5′-CGTTTCCTTTGTTCTGGA-3′)] was synthesized *in-house* according to DMT*off*-based protocols. A commercially available cationic lipid (Lipofectamine2000) was purchased from Invitrogen (Waltham, MA, USA). DMEM-Dulbecco’s modified eagle medium, trypsin-EDTA, fetal bovine serum (FBS) and distilled water (DNAse/RNAse free) were purchased from Thermo-Fischer (Waltham, MA, USA). DMEM-Dulbecco’s modified eagle medium was supplemented with 10% FBS, which was used in the experiments involving cells. The dual-luciferase reporter assay system was purchased from Promega (Madison, WI, USA) and silencing values were measured in a Promega Glomax Multidetection System instrument. Flow cytometer analyses were performed on a Guava^®^ easyCyte 8HT instrument (Millipore; Burlington, MA, USA). Flowing software (version 2.5.1, University of Turku, Finland) was used as a tool to perform flow cytometry data analysis.

### 3.2. General Protocol for Derivatizing L-Threoninol with Fmoc-Lys(Boc)-OH

Fmoc-Lys(Boc)-OH (500 mg; 1.07 mmol; 1.0 eq) and EDC (245 mg; 1.07 mmol; 1.2 eq) were mixed in dichoromethane (DCM; 3 mL) and stirred for 10 min at room temperature. N-hydroxysuccinimide (NHS) (136 mg; 1.18 mmol; 1.1 eq) was added and the final mixture was stirred at room temperature overnight. The organic layer was washed with water (2 × 10 mL), brine (2 × 10 mL) and dried over anhydrous MgSO_4_. The solvent was evaporated to dryness and the resultant crude was used without further purification. NHS-lysine derivative (**1**) (605 mg; 1.07 mmol; 1.0 eq) was dissolved in dimethylformamide (DMF; 3 mL) and *L*-threoninol (103 mg; 0.979 mmol; 1.1 eq) was added. The final solution was stirred overnight at room temperature. DMF was evaporated to dryness and the final crude was purified by flash chromatography (DCM:MeOH 4%) yielding the expected LTHR-Fmoc-Lys(Boc) (**2**; 75%).

### 3.3. General Protocol for Derivatizing 1 with Alkyl Residues of Different Length (C8 and C14)

*L*-threoninol derivative (**2**) (1.0 eq) was dissolved in an acid solution made up of DCM and trifluoroacetic acid (10%) at room temperature. The final solution was stirred for 30 min. The solvent was evaporated to dryness and the resultant trifluoroacetate salt derivative, which was used in the next step without further purification. The isolated salt (1.0 eq) was dissolved in tetrahydrofurane (THF) (2 mL) and triethylamine (TEA) (2.1 eq) was added. After stirring 10 min at room temperature, an aqueous solution of 50% *w/v* AcONa (2 mL) was added dropwise under vigorous stirring. Octanoyl chloride (1.1 eq) or myristoyl chloride (1.1 eq) were added dropwise and the resultant mixture was stirred at room temperature overnight. THF was evaporated to dryness and organic layers (5 mL of DCM) were washed with brine (3 × 10 mL). The organic layers were dried under anhydrous MgSO_4_ and solvents were reduced in vacuo. Final crudes were purified by flash chromatography (DCM:MeOH 1% to 4%), yielding the expected lipophilic derivatives **3** (97%) and **4** (78%).

(9*H*-fluoren-9-yl)methyl-(1-(((2*R*,3*R*)-1,3-dihydroxybutan-2-yl)amino)-6-octanamido-1-oxohexan-2-yl)carbamate (**3**); ^1^H NMR (400 MHz, CDCl_3_) δ 7.73 (d, *J* = 7.57 Hz, 2H), 7.56 (d, *J* = 7.56 Hz, 2H), 7.36 (t, *J* = 7.43 Hz, 2H), 7.26 (m, 2H), 6.92 (broad d, 1H; NH), 5.91 (broad m, 2H; 2 NH), 4.33 (d, *J* = 7.21 Hz, 2H; CH_2_-OH), 4.23 (m, 1H; CH-OH), 4.16 (m, 2H; CO-CH_2_-CH), 3.78 (m, 3H; CH_2_-NHCO- and CO-CH-NH-CO), 3.20 (m, 1H; CH_2_-CH-NH-), 2.10 (t, *J* = 7.46 Hz, 2H; CH_2_-CH_2_), 1.53 (m, 4H; alkyl chain), 1.23 (m, 12H; alkyl chain), 1.15 (d, *J* = 6.34 Hz, 3H; CH_3_-CH-OH), 0.84 (t, *J* = 6.67 Hz, 3H; CH_3_-CH_2_); ^13^C NMR (125 MHz, CDCl_3_) δ 174.3 (CO), 172.3 (CONH), 156,2 (COO), 143.8 (C_arom_), 143.7 (C_arom_), 141.2 (CH_arom_), 127.7 (CH_arom_), 127.0 (CH_arom_), 125.0 (CH_arom_), 119.9 (CH_arom_), 68.8 (HC-CH_2_-O), 67.0 (CH_2_-O), 64.6 (CH-O), 55.0 (OC-CH-NH), 47.1 (CH_2_-NH), 47.0 (CH_2_-CO), 39.4 (CH-NH), 38.6 (CH_2_, alkyl chain), 36.7 (CH_2_, alkyl chain), 32.3 (CH_2_, alkyl chain), 31.6 (CH_2_, alkyl chain), 29.3 (CH_2_, alkyl chain), 29.1 (CH_2_, alkyl chain), 28.9 (CH_2_, alkyl chain), 25.7 (CH_2_, alkyl chain), 22.5 (CH_2_, alkyl chain), 22.2 (CH_2_, alkyl chain), 20.2 (CH_3_-CH), 14.0 (CH_3_-CH_2_); HRMS (ESI+): *m/z* calcd for C_66_H_95_N_6_O_12_ [(2M+H)^+^] 1163.7009 found 1163.7002.

(9*H*-fluoren-9-yl)methyl-(1-(((2*R*,3*R*)-1,3-dihydroxybutan-2-yl)amino)-6-octanamido-1-oxo-6-tetradecanamidohexan-2-yl)carbamate (**4**); ^1^H NMR (400 MHz, CDCl_3_) δ 7.73 (d, *J* = 7.51 Hz, 2H), 7.58 (d, *J* = 7.42 Hz, 2H), 7.37 (t, *J* = 7.44 Hz, 2H), 7.28 (m, 2H), 6.90 (broad d, 1H; NH), 5.83 (broad m, 2H; 2 NH), 4.35 (d, *J* = 7.01 Hz, 2H; CH_2_-OH), 4.18 (m, 3H; CO-CH_2_-CH and CH-OH), 3.80 (m, 3H; CH_2_-NHCO- and CO-CH-NH-CO), 3.09 (m, 5H; CH_2_-NH, CH-NH and CH_2_-CO), 2.30 (broad s), 2.13 (t, *J* = 7.98 Hz, 2H; CH_2_-CH_2_), 1.34 (t, *J* = 7.28 Hz, 2H; CH_2_-CH_2_), 1.22 (m, 22H; alkyl chain), 1.14 (d, *J* = 6.09 Hz, 3H; CH_3_-CH-OH), 0.85 (t, *J* = 6.69 Hz, 3H; CH_3_-CH_2_); ^13^C NMR (125 MHz, CDCl_3_) δ 174.4 (CO), 172.1 (CO), 143.8 (C_arom_), 143.7 (C_arom_), 141.2 (CH_arom_), 127.7 (CH_arom_), 127.0 (CH_arom_), 125.1 (CH_arom_), 119.9 (CH_arom_), 69.2 (HC-CH_2_-O), 67.0 (CH_2_-O), 65.0 (CH-O), 54.8 (OC-CH-NH), 47.0 (CH_2_-NH), 45.7 (CH-NH), 39.2 (OC-CH_2_, alkyl chain), 38.5 (CH_2_, alkyl chain), 36.7 (CH_2_, alkyl chain), 32.2 (CH_2_, alkyl chain), 31.8 (CH_2_, alkyl chain), 29.6 (CH_2_, alkyl chain), 29.4 (CH_2_, alkyl chain), 29.2 (CH_2_, alkyl chain), 25.7 (CH_2_, alkyl chain), 22.6 (CH_2_, alkyl chain), 20.2 (CH_2_, alkyl chain), 14.0 (CH_3_-CH), 8.5 (CH_3_-CH_2_); HRMS (ESI+): *m/z* calcd for C_39_H_59_N_3_NaO_6_ [(M+Na)^+^] 688.4294 found 688.4296; *m/z* calcd for C_78_H_118_N_6_NaO_12_ [(2M+Na)^+^] 1353.8704 found 1353.8700.

### 3.4. General Protocol for a Selective Primary Alcohol Protection Using a Trityl Unit as a Protecting Group

Alcohol derivatives (**3**) and (**4**) (1.0 eq, each) and DMAP (0.5 eq) were dissolved in pyridine (1.0 mL). DIPEA (2.0 eq) was added dropwise. Reactions were stirred 5 min at room temperature and trityl chloride (TrCl) (1.6 eq) was added. Reactions were heated at 45 ℃and stirred overnight. Additional TrCl (0.5 eq) was added so that the remaining alcohols reacted completely. Reactions were stirred one more hour at room temperature. Finally, the solvent was evaporated to dryness and the resulting crudes were purified by flash chromatography (DCM to DCM:MeOH 4%), yielding the expected trityl derivatives **5** (24%) and **6** (53%).

(9*H*-fluoren-9-yl)methyl-(1-(((2*R*,3*R*)-3-hydroxy-1-(trityloxy)butan-2-yl)amino)-6-octanamido-1-oxohexan-2-yl)carbamate (**5**); ^1^H NMR (400 MHz, CDCl_3_) δ 7.74 (d, *J* = 7.57 Hz, 2H), 7.56 (d, *J* = 7.21 Hz, 2H), 7.49 (d, *J* = 7.50 Hz, 2H), 7.37 (m, 7H), 7.26 (m, 8H), 7.21 (m, 2H), 6.78 (broad d, NH), 5.65 (broad d, NH), 5.55 (broad m, NH), 4.31 (d, *J* = 7.03 Hz, 2H; CO-CH_2_-CH), 4.19 (m, 1H; CH-OH), 4.13 (m, 1H; CH-OH), 4.01 (m, 1H; CH-OH), 3.92 (m, 2H; CO-CH_2_-CH), 3.38 (m, 1H; CO-CH-NH-CO), 3.19 (m, 3H; CH_2_-NHCO- and CH-NH), 2.07 (t, *J* = 7.44 Hz, 2H; CH_2_-CH_2_), 1.54 (m, 4H; alkyl residue), 1.39 (m, 2H; alkyl residue), 1.22 (m, 10H; alkyl residue); 1.07 (d, *J* = 6.36 Hz, 3H; CH_3_-CH), 0.83 (t, *J* = 6.58 Hz, 3H; CH_3_-CH_2_); ^13^C NMR (125 MHz, CDCl_3_) δ 173.4 (CO), 172.0 (CO), 156.4 (COO), 143.3 (C_arom_), 141.2 (C_arom_), 128.4 (CH_arom_), 127.9 (CH_arom_), 127.6 (CH_arom_), 127.2 (CH_arom_), 127.0 (CH_arom_), 125.1 (C_arom_), 125.0 (C_arom_), 119.9 (CH_arom_), 119.8 (CH_arom_), 87.1 (C_q_), 68.2 (HC-CH_2_-O), 67.1 (CH_2_-O), 64.6 (CH-O), 53.8 (OC-CH-NH), 47.0 (CH_2_-NH), 38.5 (OC-CH_2_, alkyl chain), 36.8 (CH_2_, alkyl chain), 32.0 (CH_2_, alkyl chain), 31.6 (CH_2_, alkyl chain), 29.2 (CH_2_, alkyl chain), 28.9 (CH_2_, alkyl chain), 25.7 (CH_2_, alkyl chain), 22.6 (CH_2_, alkyl chain), 22.5 (CH_2_, alkyl chain), 19.9 (CH_3_-CH), 14.0 (CH_3_-CH_2_); HRMS (ESI+): *m/z* calcd for C_52_H_61_N_3_NaO_6_ [(M+Na)^+^] 846.4460 found 846.4453.

(9*H*-fluoren-9-yl)methyl-(1-(((2*R*,3*R*)-3-hydroxy-1-(trityloxy)butan-2-yl)amino)-6-tetradecanamidohexan-2-yl)carbamate (**6**); ^1^H NMR (400 MHz, CDCl_3_) δ 7.74 (d, *J* = 7.58 Hz, 2H), 7.56 (d, *J* = 7.51 Hz, 2H), 7.48 (m, 2H), 7.36 (m, 7H), 7.26 (m, 8H), 7.20 (m, 2H), 6.83 (broad d, 1H; NH), 5.70 (broad d, 1H; NH), 5.57 (broad m, 1H; NH), 4.32 (m, 2H; CO-CH_2_-CH), 4.20 (m, 1H; CH-OH), 4.12 (m, 1H; CH-OH), 4.01 (m, 1H; CH-OH), 3.92 (m, 2H; CO-CH_2_-CH), 3.37 (m, 1H; CO-CH-NH-CO), 3.19 (m, 3H; CH_2_-NHCO- and CH-NH), 2.07 (t, *J* = 7.40 Hz, 2H; CH_2_-CH_2_), 1.70 (m, 2H), 1.52 (m, 6H), 1.37 (m, 2H), 1.21 (m, 16H), 1.05 (d, *J* = 6.33 Hz, 3H; CH_3_-CH), 0.86 (t, *J* = 6.67 Hz, 3H; CH_3_-CH_2_); ^13^C NMR (125 MHz, CDCl_3_) δ 173.4 (CO), 172.1 (CO), 156.4 (COO), 143.3 (C_arom_), 141.2 (C_arom_), 128.4 (CH_arom_), 127.9 (CH_arom_), 127.6 (CH_arom_), 127.4 (CH_arom_), 127.3 (C_arom_), 127.0 (CH_arom_), 125.0 (C_arom_), 119.9 (CH_arom_), 87.0 (C_q_), 68.1 (HC-CH_2_-O), 67.1 (CH_2_-O), 64.6 (CH-O), 53.9 (OC-CH-NH), 47.0 (CH_2_-NH), 38.6 (OC-CH_2_, alkyl chain), 36.8 (CH_2_, alkyl chain), 31.8 (CH_2_, alkyl chain), 29.7 (CH_2_, alkyl chain), 29.6 (CH_2_, alkyl chain), 29.4 (CH_2_, alkyl chain), 29.3 (CH_2_, alkyl chain), 29.0 (CH_2_, alkyl chain), 25.7 (CH_2_, alkyl chain), 22.6 (CH_2_, alkyl chain), 19.9 (CH_3_-CH), 14.0 (CH_3_-CH_2_); HRMS (ESI+): *m/z* calcd for C_58_H_73_N_3_NaO_6_ [(M+Na)^+^] 930.5440 found 930.54423; *m/z* calcd for C_116_H_147_N_6_O_12_ [(2M+H)^+^] 1816.1045 found 1816.1072.

### 3.5. General Protocol for CPG Functionalization and DNA Synthesis

Trityl derivatives (1.0 eq), succinic anhydride (1.5 eq) and DMAP (1.5 eq) were dissolved in DCM (1.5 mL). The resultant solution was stirring at room temperature overnight. The organic layer was washed with a 0.5M phosphate solution. The organic layer was dried over anhydrous MgSO_4_ and the resultant hemisuccinate crudes were used in the next step without further purification. Finally, CPG functionalization was carried out according to the literature [43], affording the corresponding CPG-**7** and CPG-**8**, respectively. 3′-DNA synthesis was carried out and enabled the introduction of three distinct sequences: (1) d(TGGGGT; TG_4_T), (2) d(TGGGGGGT; TG_6_T) and (3) a 18-mer phosphorothioate oligonucleotide of sequence 5-CGTTTCCTTTGTTCTGGA-3 (*Luc*) which modified the 5′-termini of (1) and (2). After heating up the corresponding CPG solid supports at 55 °C in aqueous ammonia, unmodified oligonucleotides and families of oligonucleotide conjugates modified with lipids were isolated and purified by semi-preparative HPLC: (1) unmodified d(TGGGGT; TG_4_T, **9**) and d(TGGGGGGT; TG_6_T, **10**) oligonucleotides; (2) oligonucleotide conjugates modified with lipids at the 3′-termini; TG_4_T_C8 (**11**), TG_4_T_C14 (**12**), TG_6_T_C8 (**13**) and TG_6_T_C14 (**14**), and (3) Lipid oligonucleotide antisense conjugates; *Luc*_TG_4_T_C8 (**15**), *Luc*_TG_4_T_C14 (**16**), *Luc*_TG_6_T_C8 (**17**) and *Luc*_TG_6_T_C14 (**18**) as well as *Luc*_TG_4_T (**19**) and *Luc*_TG_6_T (**20**), which were used as controls in transfection experiments.

### 3.6. General Protocol for Introducing Aminolipids on Solid-Phase

CPG solid supports containing d(TGGGGT) and d(TGGGGGGT) as building blocks were modified with the *Luc* oligonucleotide sequence. A protected carboxylate modifier phosphoramidite with 2-chlorotrityl as a protecting group (Carboxy-C5) was automatically introduced by modifying the 5′-termini of the final sequence giving rise to the following sequences: (1) d(5-carboxylate-*Luc*-TGGGGT-3) and (2) d(5-carboxylate-*Luc*-TGGGGGGT-3). The 2-chlorotrityl protecting group was removed from using a 2% TCA solution and the resultant carboxylate group was activated with a mixture based on PyBOP (20 μmol) and HOBt (20 μmol) using DMF as an organic solvent. After 30 min at 35℃ TEA (20 μmol) and the selected amino lipids either (octylamine or tetradecylamine; 20 μmol each) in DMF were put in contact with the solid supports. Modified CPG resins were then shaked for two hours at 35 ℃ and finally washed with 500 μL of DMF, MeOH and Et_2_O. 5′-LOC were detached from the CPG solid supports in accordance with the same experimental protocols described above obtaining the following conjugates: 5′_C8_*Luc*_TG_4_T (**21**), 5′_C14_*Luc*_TG_4_T (**22**), 5′_C8-*Luc*_TG_6_T (**23**) and 5′_C14-*Luc*_TG_6_T (**24**).

### 3.7. General Protocol for the G-Quadruplex Formation

Unmodified and modified G-quadruplex nanostructures were prepared from the corresponding lyophilized oligonucleotides described in Section 2.5. Unmodified and modified oligonucleotide conjugates (**9–24**) were dissolved either in 10 mM lithium cacodylate buffer (pH 7.2) supplemented with 0.11 M NaCl or 10 mM phosphate buffer solution (pH 7.4) depending on the experiment to afford the expected G-quadruplex nanostructures [G4-(**9–24**)]. Final solutions were heated up at 95 ℃ for 5 min and slowly cooled down to room temperature. Tempered solutions were remained for five days at room temperature and finally stored at −20 °C before usage. The following G-quadruplex nanostructures were obtained: (i) unmodified G4_**9** and G6_**10**; (ii) G-quadruplex forming oligonucleotide conjugates modified at the 3′-termini with two lipid derivatives, 3′_G4_C8 (**11**), 3′_G4_C14 (**12**), 3′_G6_C8 (**13**), 3′_G6_C14 (**14**); (iii) G-quadruplex forming oligonucleotide conjugates bearing an antisense oligonucleotide (*Luc*) at the 5′-termini: 5′_*Luc*_G4_C8 (**15**), 5′_*Luc*_G4_C14 (**16**), 5′_*Luc*_G6_C8 (**17**), 5′_*Luc*_G6_C14 (**18**), *Luc*_G4 (**19**) and *Luc*_G6 (**20**) and (iv) G-quadruplex forming oligonucleotide conjugates with the 5′-termini of *Luc* sequence modified with two saturated lipids of different lengths (C8 and C14, respectively): 5′_C8_*Luc*_G4 (**21**), 5′_C14_*Luc*_G4 (**22**), 5′_C8_*Luc*_G6 (**23**) and 5′_C14_*Luc*_G6 (**24**).

### 3.8. CD Spectroscopy and Melting Experiments

The CD spectra of unmodified G-quadruplexes [d(TG_4_T)]_4_ and [d(TG_6_T)]_4_ (1.0 OD), as well as modified G-quadruplexes [G4-(**9–14**)] (1.0 OD), were registered, ranging from 220 and 320 nm at 25 ºC in the appropriate buffer (10 mM sodium cacodylate buffer, pH 7.2, supplemented with 0.11 M NaCl). CD thermal denaturation experiments were carried out using a selected range of temperatures between 20 and 80 °C and a heating rate of either 1 or 5.0 °C min^−1^. The corresponding CD values were monitored at 263 nm.

### 3.9. Electromobility Shift Assay

A native polyacrylamide gel electrophoresis (PAGE) was carried out using acrylamide concentrations of 20% and 12% (*v*/*v*) to characterize quadruplex structures (**9–25**) dissolved in PBS. A 20% PAGE gel was carried out in order to identify the different nucleic acid structures made up of 6 to 8 nucleotides (Figure 5A). On the other hand, a 12% PAGE gel was used for the characterization of larger oligonucleotides (Figure S1). 1X TBE buffer supplemented with 100 mM KCl was used to run the gels at 100 V (20% PAGE) and 150 V (12% PAGE) for approximately 4–5 h, maintaining a fixed temperature of 20 °C. A “stains-all” solution (0.001% dissolved in a mixture of formamide/H_2_O (45 mL/55 mL)) was used to stain a PAGE 20% gel for 20 min at room temperature. Finally, a picture of the gel was taken under white light. For the 12% PAGE gels, SYBR green (20 µL in 200 µL 1X TBE) was used to stain the DNA bands and then pictures were taken using Fujifilm LAS-1000 Intelligent Dark Box II as well as IR LAS-1000 Lite v1.2. In both cases, the ladder used was a solution containing Bromophenol Blue and Xylene Cyanol for visual tracking of oligonucleotides migration during the electrophoretic process.

### 3.10. Fluorescence Spectroscopy Experiments

Modified G-quadruplexes [G4_(**11–14**)] as well as unmodified [G4_**9** and G6_**10**], were dissolved in 10 mM phosphate buffer solution (pH 7.4) to obtain a final concentration of 69 nM. Fluorescence was registered using a wavelength ranging from 450–650 nm at room temperature (RT). A benzothiazole salt, like Thioflavin T (ThT), was used as fluorescence dye that interacts with the pre-formed tetrads and turns out an emission peak at the λ = 490–492 nm after ThT was sequentially added to the sample solution [48,49]. This technique is a visual tool to determine the presence of parallel G-quadruplex secondary conformation on some DNA sequences.

### 3.11. MTT Assay

Cytotoxicity experiments were studied according to the literature [52]. Cellular viabilities were carried out on a 96-well plate in HeLa cells (5·10^3^ cells/well), which were incubated in DMEM (200 μL) at 37 ℃. Both oligonucleotide conjugates (**15–24**) and G-quadruplex forming oligonucleotides [G4_(**15–24**)] were incubated at growing concentrations (60, 120 and 300 nM) for 24 h. DMEM was removed and wells were replaced with fresh DMEM (200 μL). Cells were incubated 4 more hours and MTT (3-(4,5-dimethylthiazol-2-yl)-2,5-diphenyltetrazolium bromide) was added (25 μL; 5 mg·mL^−1^). After 2 h of incubation, the medium was discharged, and formazan crystals were dissolved in DMSO (200 μL). The resulting solutions were shaken for 15 min at room temperature and cellular viability was registered at 570 nm. G4_**19** and G4_**20** were used as negative controls. Normalized data displayed the relationship between treated and non-treated cells. Cytotoxicity studies were carried out six times in two independent experiments.

### 3.12. Gene Transfection Studies

Cancerous cervical cells (HeLa) were passaged in order to maintain exponential growth. HeLa cells were seeded (10^5^ cells/well) and incubated at 37 ºC in a 24-well plate to get 60% confluence using DMEM supplemented with 10% FBS. Two different transfection experiments were carried out: (i) in the presence and (ii) in the absence of lipofectamine.

#### 3.12.1. Transfection Studies in the Presence of Lipofectamine

In the first case, *Renilla* (pRL-TK; 0.1 μg) and Firefly (pGL4; 1.0 μg) luciferase were used as reporter and control plasmids, respectively. These plasmids were combined either with oligonucleotide conjugates (**15–24**) or G4-forming oligonucleotide conjugates [G4_(**15–24**)] at 60 nM and lipofectamine (1.3 μL) in Opti-MEM. The resultant formulation was added to each well resulting in a final volume of 600 μL. Liposomes were incubated for 15 h at 37 °C. DMEM was removed and cells were washed with PBS. Finally, luciferase expression was measured according to the manufacturer’s protocol. Transfection experiments were carried out in triplicate in three independent experiments. *Luc* oligonucleotide, G4_**19** and G4_**20** were used as negative controls. The same experimental protocol was carried out as described above when G-quadruplex forming oligonucleotide **25** and T-rich oligonucleotide **26** were used at the same concentration (60 nM).

#### 3.12.2. Transfection Studies in the Abscene of Lipofectamine

*Renilla* (pRL-TK; 0.1 μg) and Firefly (pGL4; 1.0 μg) luciferase were previously formulated in liposomes using lipofectamine (1.3 μL) in Opti-MEM according to standard protocols. After 4 h of incubation, DMEM was removed and cells were washed with PBS (2 × 300 μL). Different concentrations of G-quadruplex forming oligonucleotides (**15–24**) were prepared (60, 120 and 300 nM) and used for the transfection process in 600 μL of DMEM. After being the samples incubated for 24 h, DMEM was removed and wells were thoughtfully with PBS (3 × 300 μL). Luciferase expression was measured according to the manufacturer’s protocol. Transfection experiments were carried out in triplicate in three independent experiments. *Luc* oligonucleotide, G4_**19** and G4_**20** were used as negative controls. The same experimental protocol was carried out, as described above when G-quadruplex forming oligonucleotide **25** and T-rich oligonucleotide **26** were used at the same concentration (60 nM).

### 3.13. Flow Cytometry

HeLa cells (10^5^ cells/well) were seeded in a 24-well plate the day before. Transfection experiments were performed according to the experimental procedure described before. A fluorescently labelled G4-quaduplex forming oligonucleotide conjugate was used at a concentration of 300 nM to promote a transfection process in the absence of lipofectamine. The number of positive cells was analyzed after 24 h incubation. DMEM was removed and cells were firstly washed with PBS (3 × 300 μL) and then 200 μL of EDTA-Trypsin were added. Cells containing trypsin were incubated at 37 °C for 5 min. DMEM was added (800 μL) and trypsinized cells were centrifuged ((3.0 rcf × 3 min), 8 min). Cellular pellets were suspended in PBS (500 μL) and analyzed using a flow cytometer instrument and collecting 10^4^ events in a selected gate (R1), which corresponds to the cellular population. A second gate (R2) was selected to evaluate the number of positive cells of each transfection process. The Flowing Software 2.5.1 developed by the University of Turku (Finland) was used to measure the relationship between untreated cell and positive cell populations.

### 3.14. Statistical Analysis

For in vitro transfections of G-quadruplex forming oligonucleotide conjugates, a 2-way ANOVA analysis combined with the Bonferroni post-test was used for studying statistical differences.

## 4. Conclusions

The use of covalent strategies has been addressed to modify either the 3′- or 5′-termini of a G-rich sequence with a threoninol-based scaffold containing two saturated lipids with different lengths and a phosphorothioate (*Luc*) oligonucleotide, respectively. In addition, another family of G-rich lipid oligonucleotide conjugates was prepared by solid-phase strategies involving a carboxylate modifier and two amino lipids, namely octylamine and tetradecylamine. After the preparation of the two series of lipid oligonucleotide conjugates, G-rich sequences were self-assembled in phosphate saline buffer to give rise to the corresponding modified G-quadruplex constructs. Interestingly, the presence of modifications introduced at the 3′-termini of nanostructures containing both four and six G-quartets did not disrupt the G-quadruplex stability causing their melting temperature (T_1/2_) to increase. Unfortunately, the addition of the antisense oligonucleotide to the G-quadruplexes disrupts the G-quadruplex stability, as observed by the presence of two bands in gel electrophoresis especially for the TG4T and the 5′-modified sequences.

Those modified conjugates made up of *Luc* oligonucleotide and hydrophobic pendent groups were not toxic and proved to be efficient in knocking down mRNA *Renilla* luciferase when combined with a commercially available cationic lipid. Remarkably, the conjugates containing four G-quartets and modified with 3′-threoninol pendent groups were the most potent constructs of the two antisense families prepared in this study. Transfection experiments carried out in the absence of lipofectamine revealed moderate inhibition activities when targeting *Renilla* luciferase being the oligonucleotides **19** and **15** the most efficient antisense constructs of our series. The presence of binding proteins in serum like albumin, among others might be responsible for favoring the cell entry of such oligonucleotides. To confirm the affinity of conjugates to potential receptors present in cancer cells, flow cytometry analysis using HeLa and HEK293 cells were performed. As expected, a significant amount of fluorescently labelled cell populations was obtained when transfecting HeLa cells with oligonucleotide **25,** but also with a control T-rich oligonucleotide. Although we could not confirm the potential use of G-rich oligonucleotides in the enhancement of cell uptake of antisense oligonucleotides, additional studies will be performed with G-rich oligonucleotides to deliver not only short nucleic acids but other important therapeutic cargoes like small molecule drugs that may not interfere with G-quadruplex formation.

## Figures and Tables

**Table 1:** Sequences and characterization data (MALDI-TOF mass spectrometry) of oligonucleotides.

Name	Sequence	Backbone	Modification (Mod)	Mass (Calcd)	Mass (Found)	T_1/2_ Na^+^ (°C) ^d^
**9**	TG_4_T	PO	unmod.	1863	1860	59.2 ^e^
**10**	TG_6_T	PO	unmod.	2521	2519	>80
**11**	TG_4_T_*C8*	PO	3′_Thr_C8 ^a^	2284	2281	>80
**12**	TG_4_T_*C14*	PO	3′_Thr_C14 ^a^	2368	2366	>80
**13**	TG_6_T_*C8*	PO	3′_Thr_C8 ^a^	2942	2940	>80
**14**	TG_6_T_*C14*	PO	3′_Thr_C14 ^a^	3026	3025	>80
**15**	*Luc*-TG_4_T_*C8*	PS/PO	3′_Thr_C8 ^b^	8095	8081	-
**16**	*Luc*-TG_4_T_*C14*	PS/PO	3′_Thr_C14 ^b^	8179	8168	-
**17**	*Luc*-TG_6_T_*C8*	PS/PO	3′_Thr_C8 ^b^	8751	8749	
**18**	*Luc*-TG_6_T_*C14*	PS/PO	3′_Thr_C14 ^b^	8837	8838	-
**19**	*Luc*-TG_4_T	PS/PO	unmod.	7672	7365 ^f^	-
**20**	*Luc*-TG_6_T	PS/PO	unmod.	8330	8328	-
**21**	*C8*_*Luc*-TG_4_T	PS/PO	5′_C8_NH ^c^	7980	8040 ^g^	-
**22**	*C14*_*Luc*-TG_4_T	PS/PO	5′_C14_NH ^c^	8063	8100 ^g^	-
**23**	*C8*_*Luc*-TG_6_T	PS/PO	5′_C8_NH ^c^	8637	8635	-
**24**	*C14*_*Luc*-TG_6_T	PS/PO	5′_C14_NH ^c^	8722	8573 ^h^	-
**25**	F_*Luc*_TG_4_T_*C8*	PS/PO	3′_Thr_C8 ^a^	8646	8648	-
**26**	F_*Luc*_T_6__*C8*	PS/PO	3′_Thr_C8 ^a^	8546	8548	-

*Luc* sequence: d(5-CGTTTCCTTTGTTCTGGA-3); *unmod*: unmodified; PO: phosphodiester; PS: phosphorothioate (underlined); F: fluorescein. ^a^ Oligonucleotide conjugates containing hydrophobic threoninol-based derivatives with distinct length of hydrocarbon alkyl chains (C8 or C14); ^b^ LOC containing the *Luc* sequence at the 5′-end of the G-rich sequence; ^c^ LOC containing two hydrocarbon alkyl chains of distinct length (C8 or C14) at the 5′-end of the *Luc* sequence; ^d^ apparent melting temperature (non-equilibrium) of the G4 constructs in 10 mM sodium cacodylate buffer with 0.15 M NaCl (pH 7.2) with a heating rate of 1.0 °C min^−1^; ^e^ Ref. [27]; ^f^ the fragment [GGGGT_Luc]^+^ is observed [27]; ^g^ [M+ K^+^]; ^h^ [M-octylamide].

## Data Availability

The data presented in this study are available on request from the corresponding author.

## Supplementary Materials

The following are available online at https://www.mdpi.com/1422-0067/22/1/121/s1, ^1^H-NMR and ^13^C-NMR spectra; Figure S1. Native PAGE of antisense G-quadruplex conjugates. Figure S2: ThT assay; Table S1: Affinity constants; Figure S3. Cytotoxicity analysis on HEK2983 cells; Figure S4: Flow cytometry analysis at 60 nM; Figure S5: Flow cytometry analysis at 300 nM.
