# Supplementary material for: Tuning G-Quadruplex Nanostructures with Lipids. Towards Designing Hybrid Scaffolds for Oligonucleotide Delivery
Source: Int J Mol Sci. 2020 Dec 24;22(1):121. doi: 10.3390/ijms22010121 (PMC7796380; doi:10.3390/ijms22010121)

Supplementary material

Tuning G-Quadruplex Structures with Lipids. Towards Designing Hybrid Scaffolds for Oligonucleotide Delivery

Santiago Grijalvo [1,2], Anna Clua [1,2], Marc Eres [1], Raimundo Gargallo [3] and Ramon Eritja [1,2,*]

[1] Institute of Advanced Chemistry of Catalonia (IQAC-CSIC), Jordi Girona 18-26, E-08034 Barcelona, Spain
[2] Networking Center on Bioengineering, Biomaterials and Nanomedicine (CIBER BBN), Jordi Girona 18-26, E-08034 Barcelona, Spain
[3] University of Barcelona, Department of Chemical Engineering and Analytical Chemistry, Martí i Franquès 1-11, E-08028 Barcelona, Spain

CONTENT

Description	Page
^{1}H-NMR and ^{13}C-NMR spectra	2
Figure S1 Native PAGE of antisense G-quadruplex conjugates	7
Figure S2. ThT assay	8
Table S1. Affinity constants	9
Figure S3. Cytoxicity analysis on HEK293 cells	10
Figure S4. Flow cytometry analysis at 60 nM	11
Figure S5. Flow cytometry analysis at 300 nM	12

(9*H*-fluoren-9-yl)methyl-(1-(((2*R*,3*R*)-1,3-dihydroxybutan-2-yl)amino)-6-octanamido-1-oxohexan-2-yl)carbamate

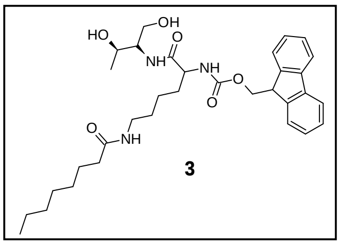

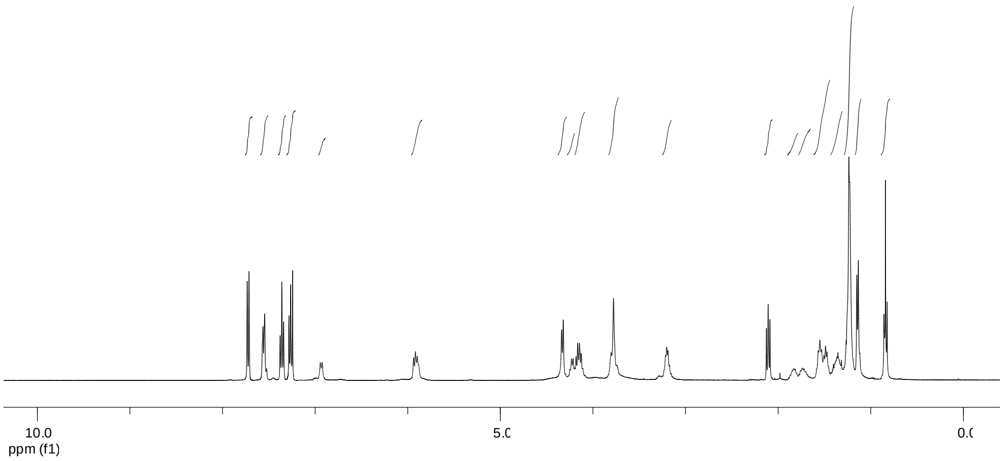

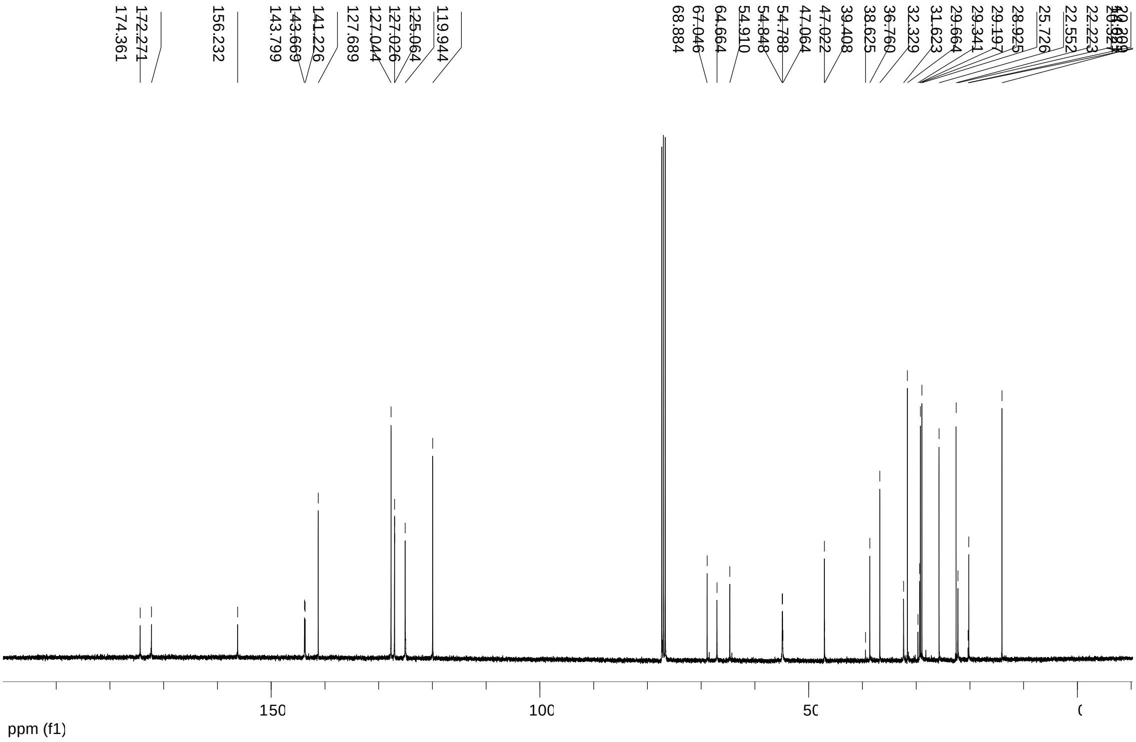

(9*H*-fluoren-9-yl)methyl-(1-(((2*R*,3*R*)-1,3-dihydroxybutan-2-yl)amino)-6-octanamido-1-oxo-6-tetradecanamidohexan-2-yl)carbamate

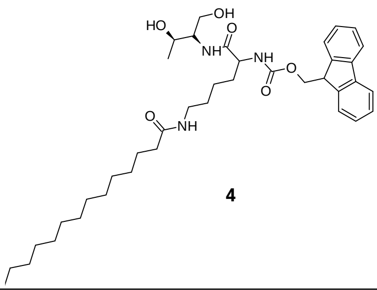

4

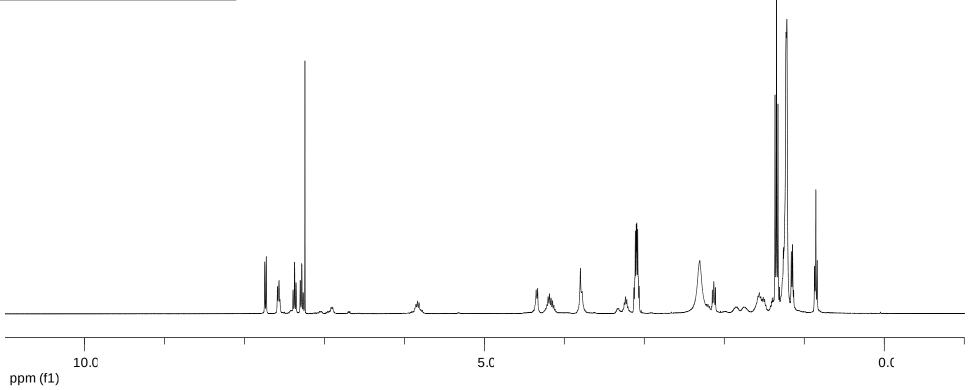

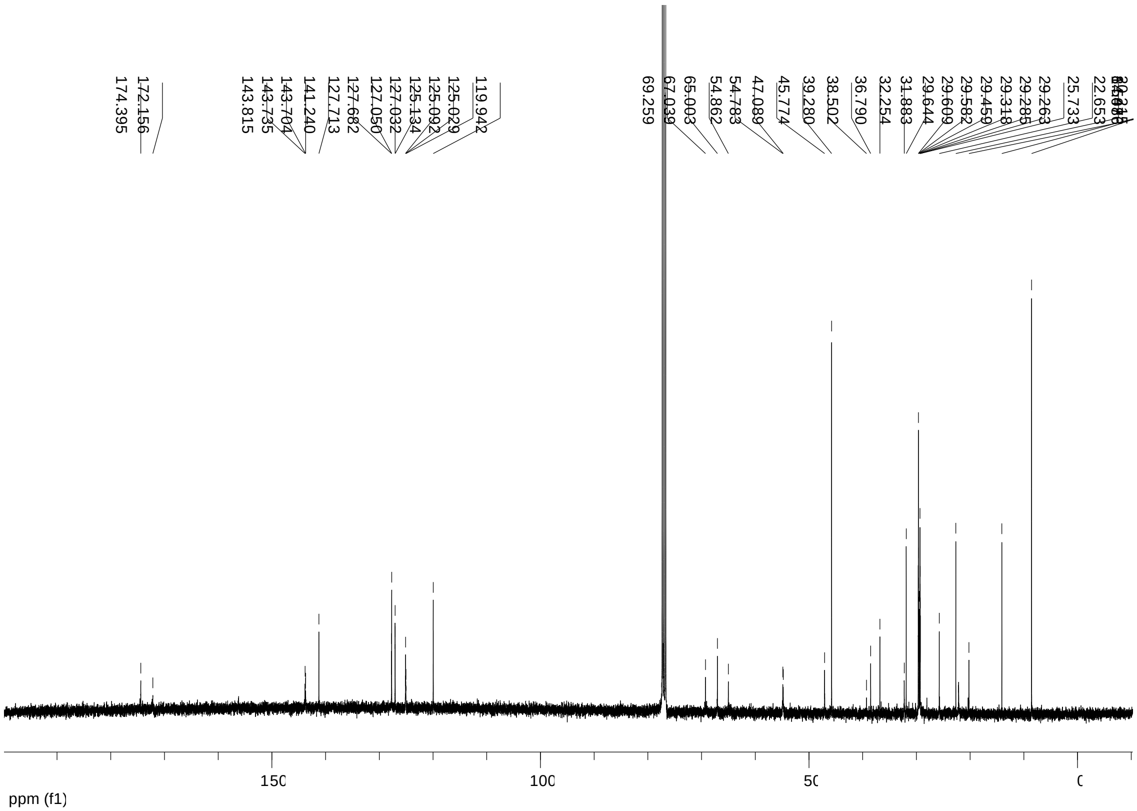

(9*H*-fluoren-9-yl)methyl-(1-(((2*R*,3*R*)-3-hydroxy-1-(trityloxy)butan-2-yl)amino)-6-octanamido-1-oxohexan-2-yl)carbamate

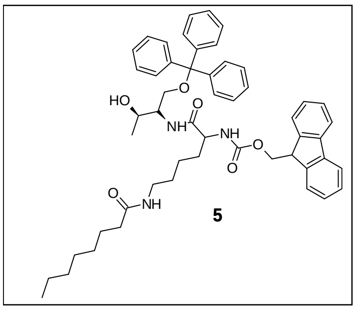

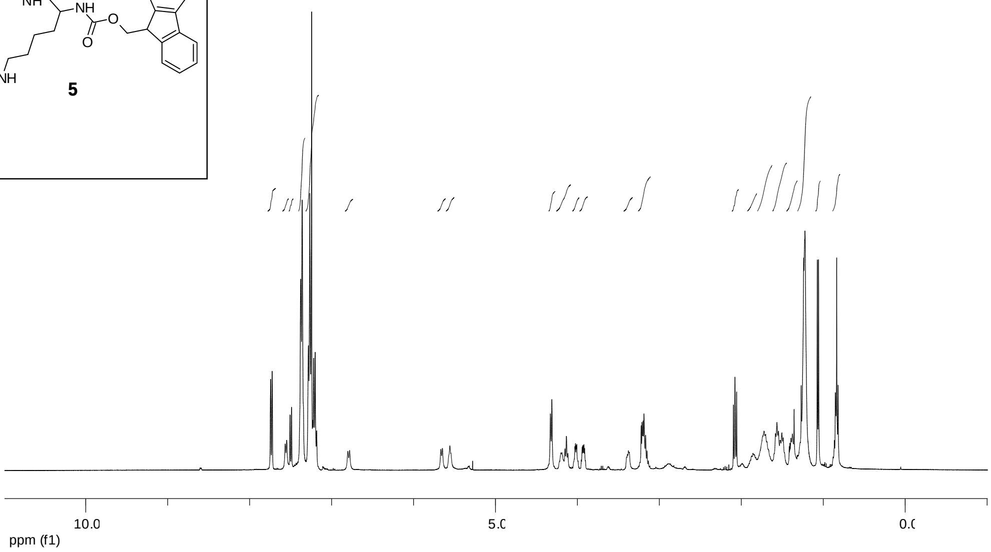

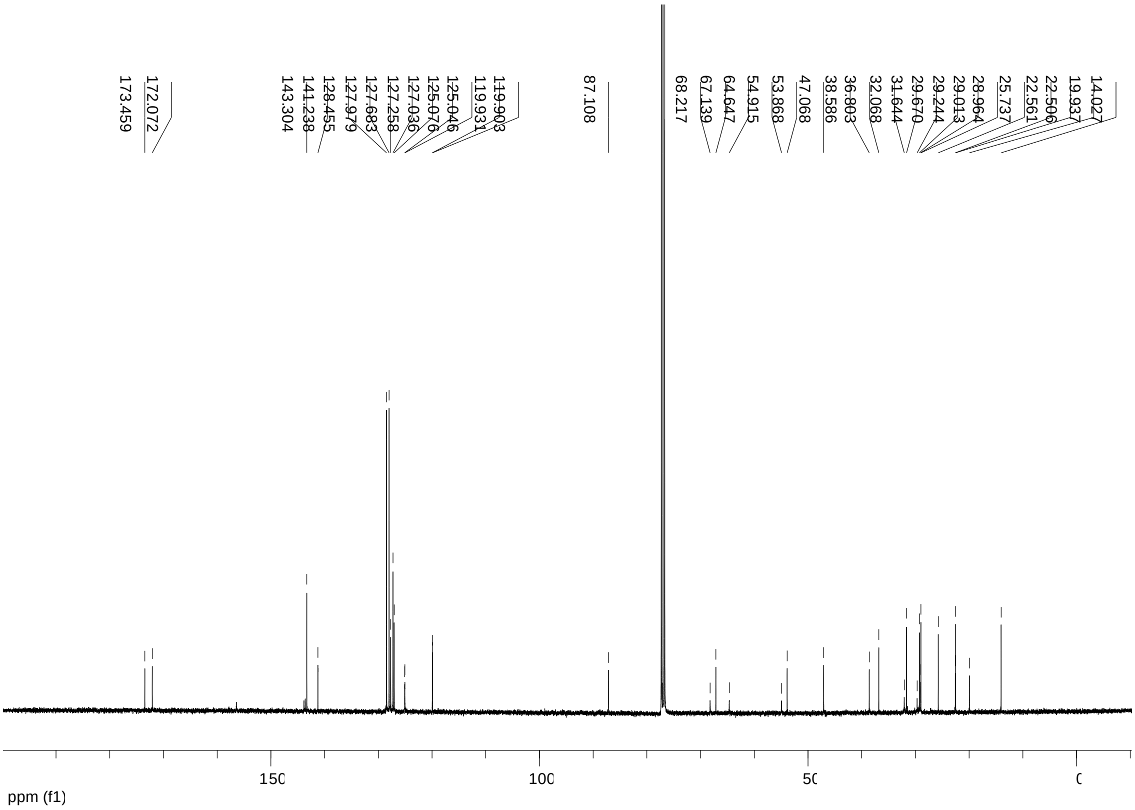

(9*H*-fluoren-9-yl)methyl-(1-(((2*R*,3*R*)-3-hydroxy-1-(trityloxy)butan-2-yl)amino)-6-tetradecanamidohexan-2-yl)carbamate

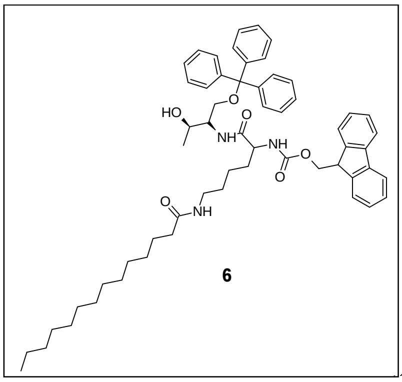

6

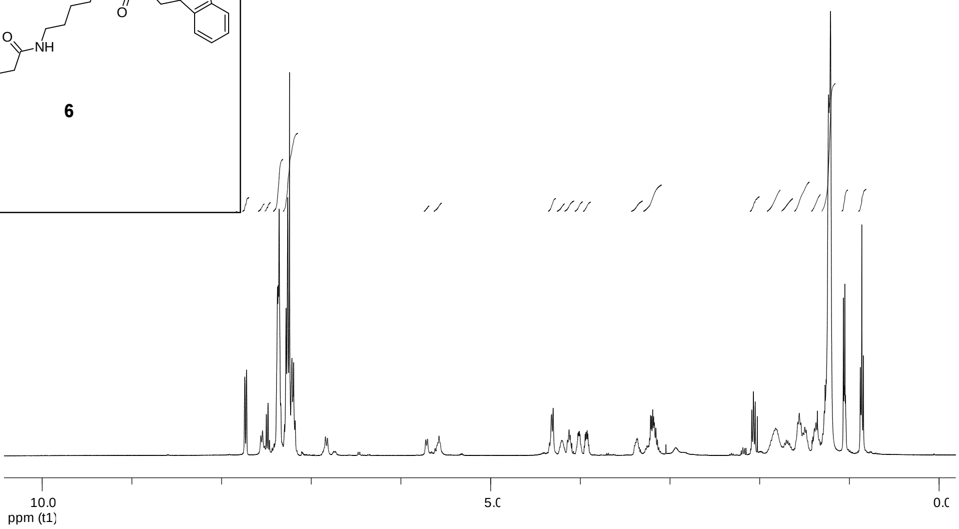

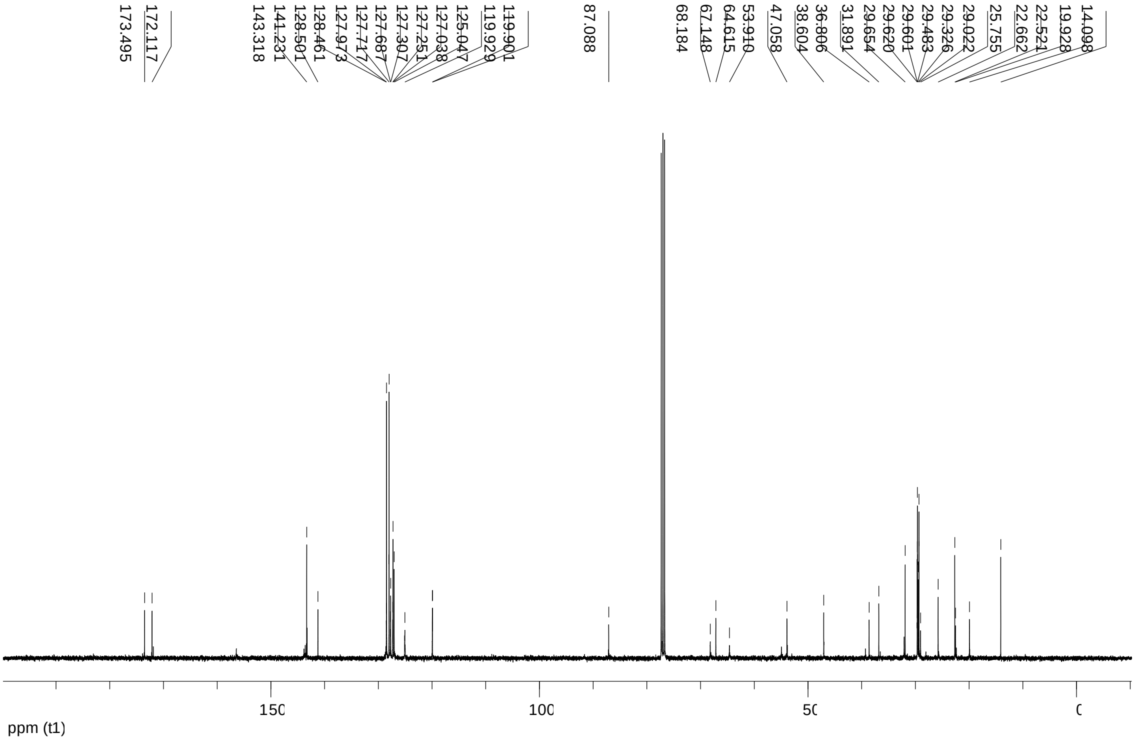

Figure S1. Native 12% polyacrylamide gel electrophoresis (PAGE) analysis of antisense G-quadruplex constructs containing A. 3'-lipid-threoninol based modifications (**15**-**20** and **24**) and B. 5'-lipid moieties (**19**-**26**). Conditions: 1X PBS supplemented with 100 mM KCl. The gel was stained with SYBR-green. The gels have been reversed to visualize the singled-stranded oligonucleotide on the top (fast runners) and the spots corresponding to quadruplex at the bottom (slow runners).

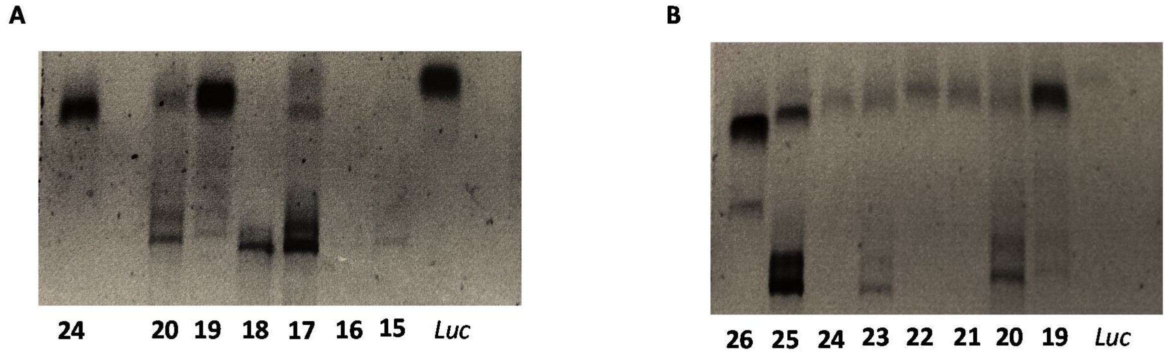

Figure S2. ThT assay using a fixed concentration of G-quadruplex (69 nM). ThT dye was added at increasing concentrations and recording its fluorescence intensity after binding to G-constructs

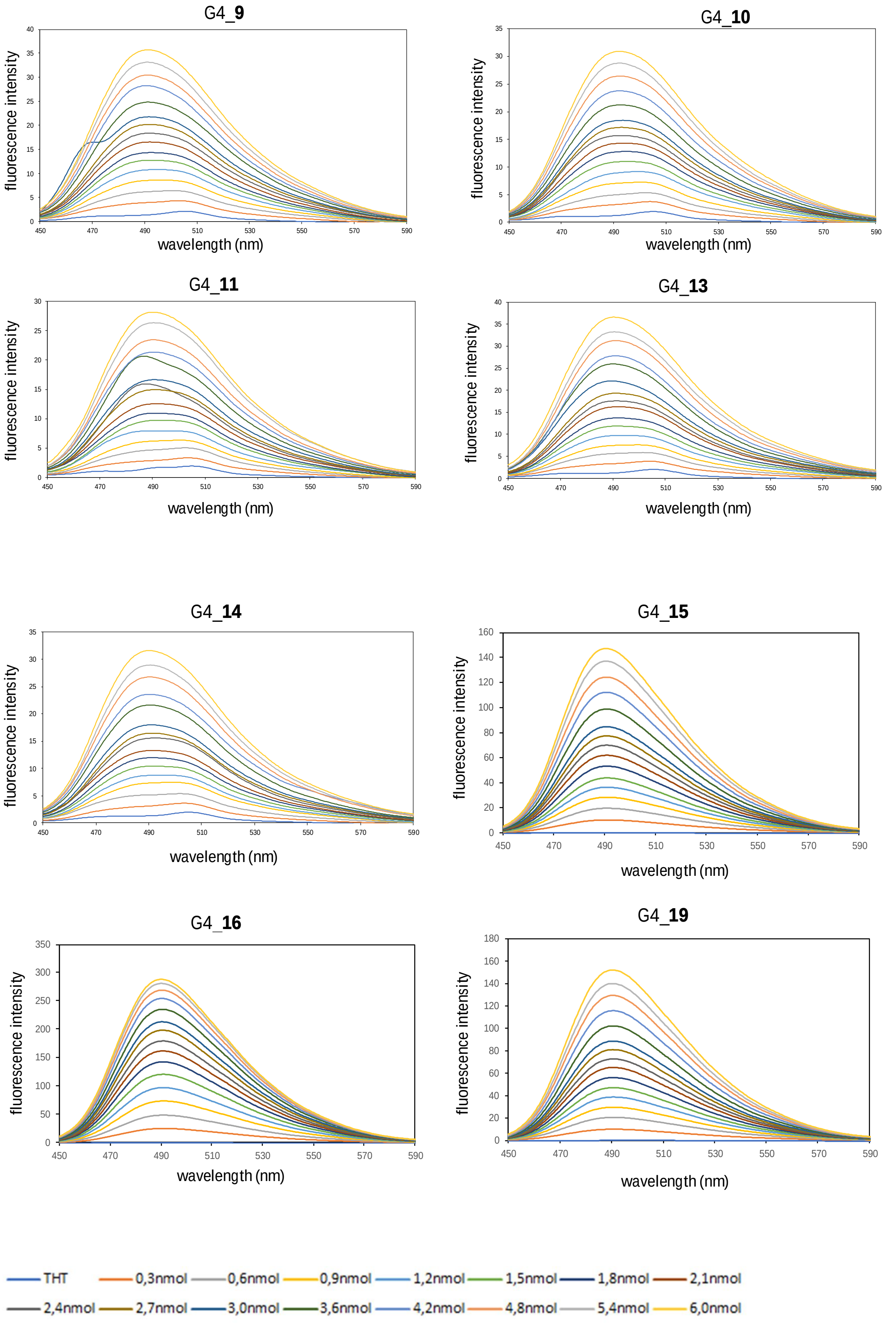

Table S1. Affinity constants of lipid oligonucleotide and LOCs by ThT fluorescence spectrometry post G-quadruplex formation

Name	Sequence	Backbone	Modification (mod)	Ka	$\sum r^2$	$\sum Fexp^2$	$\sum r^2/\sum Fexp^2$
**9**	TGGGGT	PO	unmod.	1.60E+6	64.37	69886.6	0.09%
**10**	TGGGGGGT	PO	unmod.	1.51E+6	53.78	51076	0.11%
**11**	TGGGGT_*mod*	PO	3'_Thr_C8[a]	1.41E+6	55.2	39085.3	0.14%
**13**	TGGGGGGT_ *mod*	PO	3'_Thr_C8[a]	1.40E+6	92.7	69169	0.13%
**14**	TGGGGGGT_ *mod*	PO	3'_Thr_C14[a]	1.30E+6	72.6	49417.29	0.15%
**15**	*mod* _*Luc*-TGGGGT	PS/PO	5'_*Luc*_Thr_C8[b]	1.42E+6	262	1.31E+06	0.02%
**16**	*mod* _*Luc*-TGGGGT	PS/PO	5'_*Luc*_Thr_C14[b]	4.11E+6	318.7	1211540.5	0.03%
**19**	*Luc*-TGGGGT	PS/PO	unmod.	1.44E+6	985.1	6643506.3	0.01%

Luc sequence: d(5'-CGTTTCCTTTGTTCTGGA-3'); unmod: unmodified; PO: phosphodiester; PS: phosphorothioate (underlined); [a]Oligonucleotide conjugates containing hydrophobic threoninol-based derivatives with distinct length of saturated alkyl chains (C8 or C14); [b]LOC containing the *Luc* sequence at the 5'-end of the G-rich sequence.

Figure S3. Cytotoxicity analysis on HEK293 cells of antisense G-quadruplex conjugates containing four G-tetrads and six G-tetrads (see Table 1). Four concentrations of antisense G-quadruplex conjugates ranging from 60 nM to 600 nM were used. All antisense conjugates were incubated up to 24 hours at 37 ºC. Data were means ±SD of three independent experiments

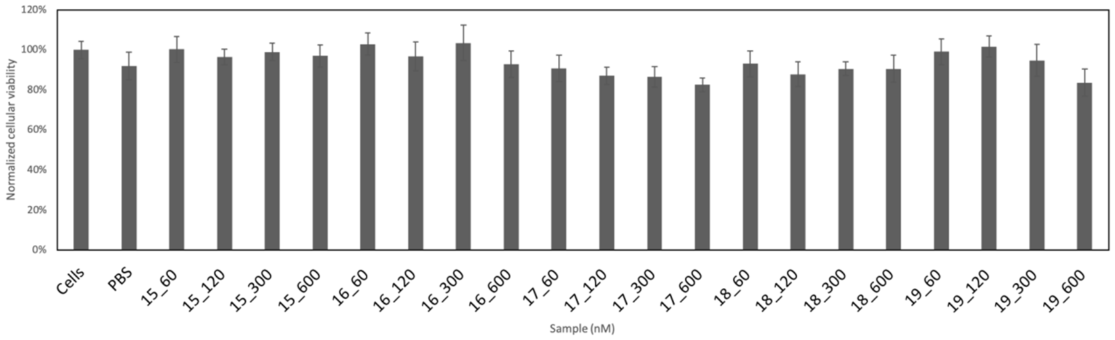

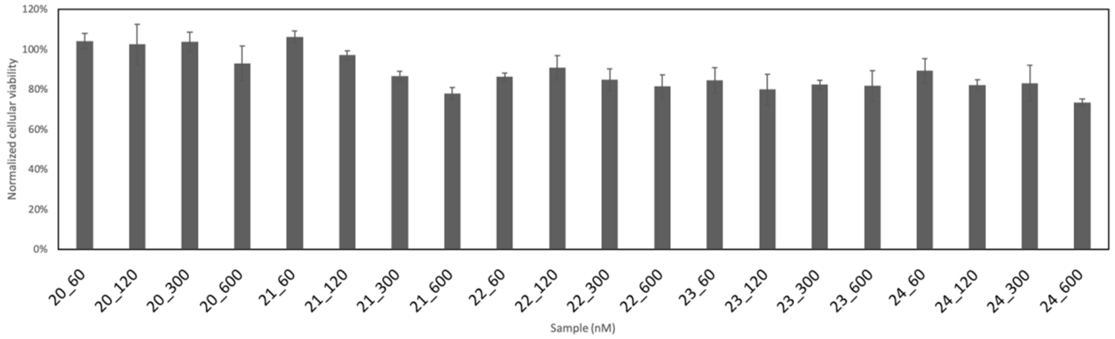

Figure S4. Flow cytometry analysis involving the effect of G4_**25** and conjugate **26** when transfecting HeLa cells at 60 nM. (A) First row: non-fluorescent labelled cell populations (Blank) (left), selected region R2 (center) and histogram of non-fluorescence cells (right); second row: forward scatter dot plot of G4_**25** (left); forward scatter dot plot of conjugate **26** (center) and histogram (right) in the presence of HeLa cells at 60 nM. The Flowing Software 2.5.1 was used to measure the relationship between untreated cell and positive cell populations.

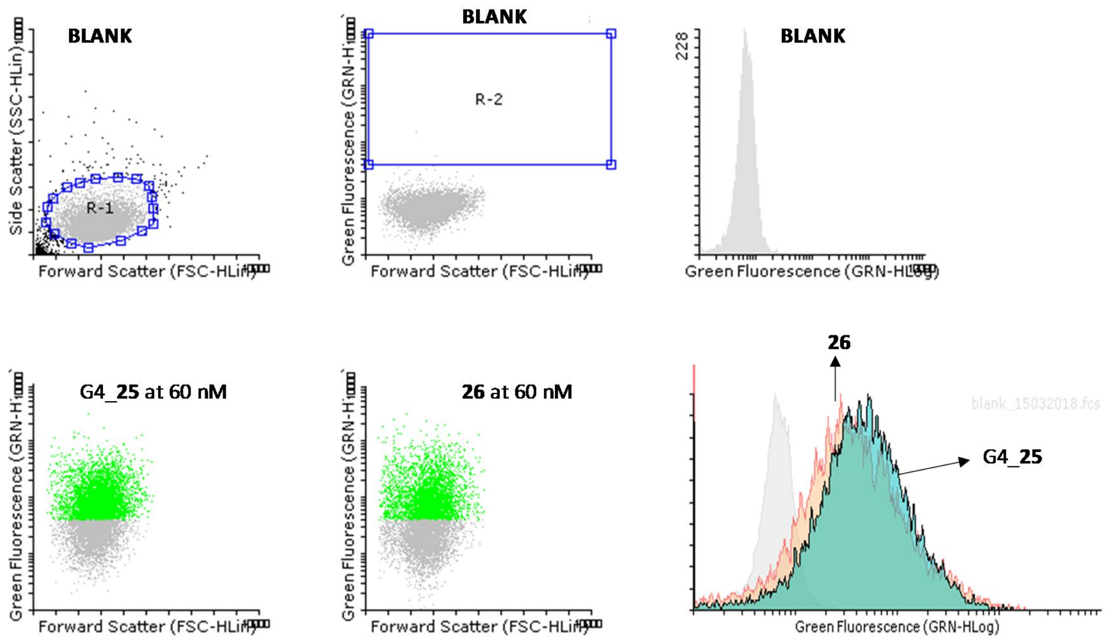

Figure S5. Flow cytometry analysis involving the effect of G4_**25** and conjugate **26** when transfecting HeLa cells at 300 nM. (A) First row: non-fluorescent labelled cell populations (Blank) (left), selected region R2 (center) and histogram of non-fluorescence cells (right); second row: forward scatter dot plot of **26** (left); forward scatter dot plot of conjugate G4_**25** (center) and histogram (right) in the presence of HeLa cells at 300 nM. The Flowing Software 2.5.1 was used to measure the relationship between untreated cell and positive cell populations.

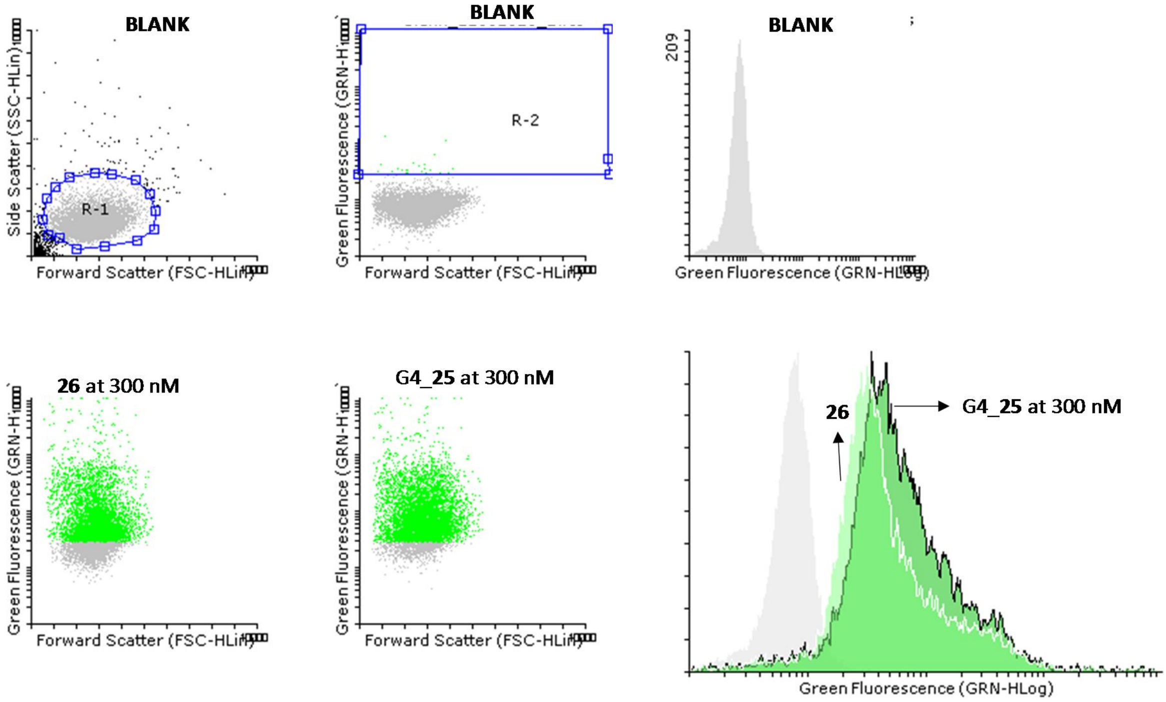

Supplement: Supplementary file 1 [file ijms-22-00121-s001.pdf]
